# MARPLE: A Proximity‐Triggered CRISPR‐Cas13 Platform for Ultrasensitive Antibody Detection

**DOI:** 10.1002/advs.202517799

**Published:** 2025-12-22

**Authors:** Elena Spezzani, Luca Capelli, Denise Di Lena, Alejandro Chamorro‐Garcia, Rudy Ippodrino, Alessandro Porchetta, Alessandro Bertucci

**Affiliations:** ^1^ Department of Chemistry Life Sciences and Environmental Sustainability University of Parma Parma Italy; ^2^ Department of Sciences and Chemical Technologies University of Rome Tor Vergata Rome Italy; ^3^ Ulisse BioMed Laboratories Trieste Italy; ^4^ Biostructures and Biosystems National Institute (INBB) Rome Italy

**Keywords:** antibody, CRISPR‐Cas13, DNA nanotechnology, immunoassay, proximity assay

## Abstract

Monitoring clinically relevant antibodies—as biomarkers of disease or therapeutic response—is essential for informed clinical decision‐making. Traditional immunoassays like ELISA offer reliable quantification but often involve multistep workflows and limited point‐of‐care utility. New approaches coupling antibody recognition with signal amplification are therefore highly desirable. The CRISPR‐Cas13 system, known for its potent collateral cleavage activity, has emerged as a powerful diagnostic tool for nucleic acid detection. However, its application to protein biomarkers such as antibodies remains underdeveloped. Here, we introduce MARPLE (Modular Antibody Recognition via Proximity‐triggered Linker Exchange), a modular CRISPR‐Cas13–based platform for ultrasensitive antibody detection. MARPLE harnesses antibody‐induced proximity to trigger a strand displacement reaction that releases a sequestered RNA target, activating Cas13‐mediated collateral cleavage of fluorescent RNA reporters. This cascade enables detection of antibodies at femtomolar concentrations. We demonstrate MARPLE's versatility across diverse targets—including anti‐digoxigenin, anti‐cholesterol, anti‐HA, trastuzumab, and anti‐MUC1—highlighting applications in infectious disease monitoring, cancer diagnostics, and therapeutic drug tracking. The assay is isothermal, one‐pot, and retains robust performance in complex matrices such as human serum. These features establish MARPLE as a promising tool for immunodiagnostics, extending CRISPR‐based sensing beyond nucleic acids to protein biomarker detection.

## Graphical abstract

MARPLE is a modular CRISPR‐Cas13 immunoassay that uses antibody‐induced proximity to trigger a toehold‐exchange reaction, releasing an RNA activator for Cas13. This one‐pot, isothermal architecture enables ultrasensitive (femtomolar) and highly specific detection of diverse antibodies, including therapeutic, pathogen‐specific, and cancer‐associated targets, with robust performance in human serum.

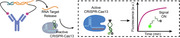

## 1 Introduction

Antibodies, glycoproteins produced by B lymphocytes, are central to adaptive immunity, recognizing and neutralizing foreign antigens [1, 2]. Abnormal antibody responses, however, can lead to autoimmune diseases and cancer through the production of autoantibodies. [3] Monoclonal antibodies have become important therapeutics for immunodeficiencies, autoimmune disorders, and cancers, but their clinical effectiveness can be limited by anti‐drug antibodies (ADAs), which affect pharmacokinetics and clearance [4, 5, 6, 7]. Given their dual role as both biomarkers and therapeutic agents, antibodies are critical targets in clinical diagnostics. Information about their presence, concentration, and bioavailability provides valuable insights into immune status, disease progression, and treatment response [8, 9, 10]. Although widely used platforms such as enzyme‐linked immunosorbent assays (ELISAs) and chemiluminescent immunoassays (CLIAs) offer high sensitivity, they typically involve multistep workflows, require substantial hands‐on time and reagents, and are not readily adaptable for rapid or point‐of‐care (POC) use. Developing alternative strategies that couple antibody recognition to signal amplification in a simple, time‐efficient, programmable, and scalable manner thus remains an important challenge. CRISPR (Clustered Regularly Interspaced Short Palindromic Repeats) systems have emerged as powerful tools for molecular diagnostics [11]. Originally discovered as a prokaryotic immune mechanism [12, 13], CRISPR systems utilize Cas (CRISPR‐associated) enzymes guided by CRISPR RNAs (crRNAs) to recognize and cleave nucleic acid targets with high specificity and programmability [13, 14, 15]. This has enabled their adaptation into highly sensitive and versatile detection platforms [11]. In particular, Cas12 and Cas13 enzymes have transformed the field of molecular diagnostics due to their unique collateral (trans‐cleavage) activity [15, 16, 17, 18]. Upon recognition of a specific DNA (Cas12) or RNA (Cas13) target, these enzymes activate nonspecific cleavage of nearby single‐stranded nucleic acids [18, 19, 20, 21]. By pairing this activity with single‐stranded nucleic acid reporters, highly sensitive assays such as DETECTR and SHERLOCK have achieved attomolar‐level detection of nucleic acid targets [21, 22]. Despite this progress, their inherent requirement for nucleic acid inputs presents a barrier to the direct use of CRISPR systems in the detection of non‐nucleic acid analytes, such as proteins. To overcome this limitation, several strategies have been proposed that convert the presence of non‐nucleic acid analytes such as proteins, small molecules, and metabolites into nucleic acid inputs compatible with CRISPR‐based readout [23, 24, 25, 26, 27, 28, 29]. Antibodies offer a unique opportunity for such transduction due to their bivalency and high‐affinity antigen‐binding properties, which can be harnessed in proximity‐based sensing systems [30, 31, 32]. DNA nanotechnology has provided a versatile toolkit for engineering such systems, integrating antigen‐antibody recognition with dynamic DNA mechanisms such as strand displacement reactions, conformational switching, or nanostructure assembly [33, 34, 35, 36, 37, 38, 39, 40]. Recently, several DNA‐based strategies have been coupled with CRISPR‐Cas systems to enable antibody detection. For instance, UCAD achieves attomolar sensitivity in human serum by using DNA‐labeled secondary antibodies to convert antibody binding into DNA products amplified by recombinase polymerase amplification (RPA), generating targets for Cas12 activation [41]. Alternatively, antigen recognition has been used to induce conformational changes in PAM‐engineered DNA constructs, exposing hidden CRISPR target sites and activating Cas12a [42]. Despite these advances, most current systems rely exclusively on Cas12 enzymes, with the application of Cas13 remaining largely underexplored. MAIGRET, an in vitro transcription‐based platform, was among the first to explore Cas13 for antibody detection. Adapted from Cas12, it detects a model antibody with picomolar sensitivity in buffer, but it does not directly link antibody binding to Cas13 activation, instead relying on transcription of the crRNA to trigger Cas13 [43]. Here, we report the first one‐pot diagnostic system for clinically relevant antibodies that directly transduces antibody‐antigen recognition into CRISPR‐Cas13–based signal amplification, without requiring auxiliary enzymes or transcription steps. We have named our assay MARPLE—*Modular Antibody Recognition via Proximity‐triggered Linker Exchange*—in the same lineage of DETECTR, SHERLOCK, and MAIGRET. Central to our design is a proximity‐based sensing module that links antibody binding to the release of an RNA target capable of activating Cas13. Specifically, antibody binding brings together two antigen‐conjugated single‐stranded DNA strands, triggering a proximity‐induced toehold‐exchange reaction. The displaced RNA target can then be recognized by CRISPR‐Cas13, activating Cas13‐mediated collateral cleavage of fluorescent RNA hairpin reporters in solution. The resulting fluorescence output is proportional to antibody concentration and enables detection down to the femtomolar range in diluted human serum. The use of the Cas13 enzyme is fundamental to our design because its trans‐cleavage activity is restricted exclusively to the RNA reporters, leaving the DNA components of the system unaffected. Thanks to its modular architecture, MARPLE can be adapted to a broad spectrum of antibody targets by simply exchanging the antigen moieties while maintaining a common detection framework. We demonstrated this versatility by detecting various antibodies, including anti‐digoxin and anti‐cholesterol as model small molecule‐binding antibodies, anti‐hemagglutinin (HA, Influenza virus) as a representative marker of immune response to pathogens, trastuzumab as a therapeutic monoclonal antibody, and anti‐mucin1 (Anti‐MUC1) as a cancer‐associated autoantibody. In all cases, MARPLE operates under isothermal, one‐pot conditions and shows robust performance in diluted human serum. By directly linking antibody recognition to Cas13 activation, MARPLE addresses an unmet need for ultrasensitive, rapid, and clinically relevant antibody detection beyond the capabilities of current immunoassays.

## 2 Results and Discussion

### 2.1 Optimization of the Proximity‐Triggered Mechanism

**FIGURE 1:**
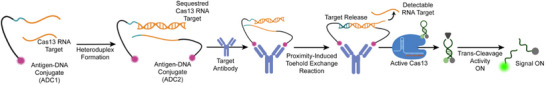
Schematic illustration of Modular Antibody Recognition via Proximity‐triggered Linker Exchange (MARPLE). Initially, a first antigen‐DNA conjugate (ADC1) holds the Cas13 RNA target sequestered within a DNA‐RNA heteroduplex. When the target antibody is present, its bivalent binding brings the ADC1‐RNA heteroduplex and a second antigen‐DNA conjugate (ADC2) into close proximity, triggering a proximity‐induced toehold exchange reaction. This reaction releases the RNA target, which then activates Cas13 trans‐cleavage activity. Activated Cas13 degrades RNA hairpin reporters in solution, producing a fluorescent signal proportional to antibody concentration.

**FIGURE 2:**
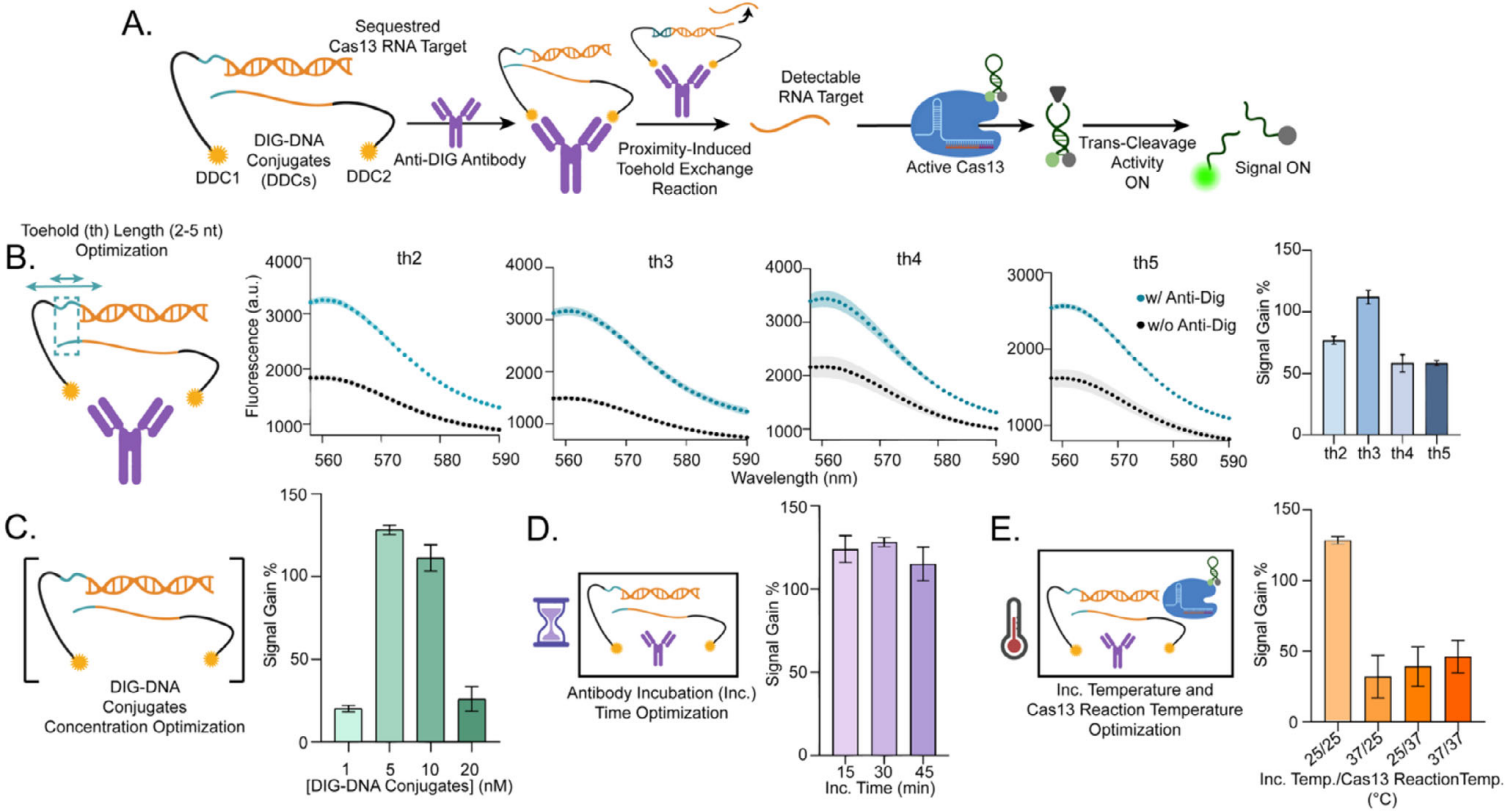
(A) Schematic representation of the MARPLE workflow for anti‐DIG antibody detection. (B) Optimization of toehold length for antibody‐induced toehold‐exchange reaction. Fluorescence spectra show Cas13 trans‐cleavage activity in the presence (light blue) or absence (gray) of anti‐DIG antibodies. (C) Optimization of nucleic acid component concentrations. Fluorescence signals from Cas13 activity were measured using varying concentrations of the DDC1–RNA target heteroduplex and DDC2, with or without anti‐DIG antibody. (D) Optimization of incubation time for antibody‐triggered RNA release. Fluorescence was recorded at different incubation times following the addition of anti‐DIG antibody to the reaction mixture. (E) Temperature optimization for one‐pot detection. Fluorescence intensities were compared using incubation and Cas13 reaction steps at either 25°C or 37°C to assess optimal conditions for antibody binding and Cas13 activity. Unless otherwise specified, all experiments were conducted in PBS containing 5 mM Mg^2^⁺, using 5 nM each of the DCD1–RNA heteroduplex and DCD2, and 10 nM anti‐DIG antibody. After a 30 min incubation at 25°C, Cas13 (30 nM) and RNA hairpin reporters (100 nM) were added, and fluorescence signals were measured after 25 min at 25°C. Signal gain (%) was calculated as the fluorescence signal increase in the presence of the antibody relative to the background. Error bars and shaded areas represent standard deviation across three independent replicates (n=3).

MARPLE leverages the unique recognition properties of antibodies and the programmability of DNA strands, which can be readily functionalized and integrated into DNA nanotechnology frameworks [44]. The Y‐shaped structure of antibodies, featuring two identical antigen‐binding sites separated by approximately 6–14 nm [45], provides a natural scaffold for inducing molecular proximity between DNA strands when each is conjugated to the same antibody target. This antibody‐induced proximity increases the effective local concentration of the DNA strands, promoting otherwise‐unfavored hybridization processes [37, 46, 47]. In our system, two antigen‐conjugated single‐stranded DNA strands (Antigen‐DNA Conjugate 1, ADC1, and Antigen‐DNA Conjugate 2, ADC2) are designed to undergo a toehold‐exchange reaction upon antibody binding. ADC1 carries a hybrid DNA–RNA duplex that sequesters the single‐stranded RNA target needed for Cas13 activation. ADC2 is capable of displacing this RNA strand through toehold exchange, forming a stable ADC1–ADC2 DNA duplex. However, this reaction is highly inefficient at low concentrations, and spontaneous strand exchange is negligible in the absence of the antibody. Only upon antibody binding, the two DNA strands are brought into sufficient proximity to enable the reaction, leading to the release of the RNA activator and subsequent formation of the ADC1–ADC2 duplex. The released RNA strand then activates Cas13, which in turn initiates collateral cleavage of RNA hairpin reporters present in solution. These reporters are labeled with a fluorophore and a quencher at opposite ends. Upon Cas13‐mediated cleavage of the single‐stranded loop region, the hairpin structure is disrupted, separating the fluorophore from the quencher and generating a fluorescent signal (Figure 1). Hairpin‐based reporters have been shown to provide superior signal‐to‐noise (S/N) ratios compared to conventional linear ones, thereby enhancing detection sensitivity [48]. We initially investigated the proximity‐triggered toehold exchange reaction using a simplified, fully DNA‐based model system, in which both antibody and antigen were mimicked by DNA constructs (Figure S1). In this setup, the displacement of a fluorophore‐labeled DNA strand was monitored to assess the efficiency of the proximity‐triggered strand exchange reaction. Using this model, we systematically varied the toehold length from 2 to 6 nucleotides and found that increasing the toehold length beyond 5 nucleotides yielded no significant improvement in signal gain (Figure S1). Based on these results, we moved on to testing the complete MARPLE assay employing anti‐digoxigenin (DIG) antibodies and DIG‐DNA conjugates (DDCs), along with the Cas13 RNA target and RNA reporters (Figure 2A). We tested toehold lengths between 2 and 5 nucleotides and found that a 3‐nucleotide toehold (th3) yielded the highest signal gain (112 % ± 5 %) relative to controls lacking antibody (Figure 2B). This condition was selected for further assay optimization. Next, we optimized the concentrations of the nucleic acid components, a key factor for proximity‐triggered reactions (Figure 2C). The concentrations of the ADC1–RNA heteroduplex and ADC2 must be sufficiently low to prevent unintended interactions and minimize background in the absence of the antibody, while still supporting efficient antigen recognition and proximity‐induced exchange in the presence of the antibody. We observed the highest signal gain (128 % ± 3 %) when both components were used at 5 nM (Figure 2C). Using this concentration, the minimum incubation time required to obtain the maximal signal gain was 15 min (Figure 2D). We further assessed the optimal temperature for achieving the best performance, as this depends on the combined contributions of multiple factors, including antigen binding, toehold exchange reaction, Cas13 activation, and reporter stability. We found that conducting the entire assay at 25°C produced the best overall performance (SG % = 111 ± 8), supporting a fully isothermal and one‐pot reaction configuration (Figure 2E). Finally, we verified the antibody‐induced strand exchange by native polyacrylamide gel electrophoresis (PAGE), which confirmed RNA release and duplex formation under the optimized assay conditions (Figure S2)

### 2.2 CRISPR‐Cas13‐Based Detection of Small Molecule‐Binding Antibodies

**FIGURE 3:**
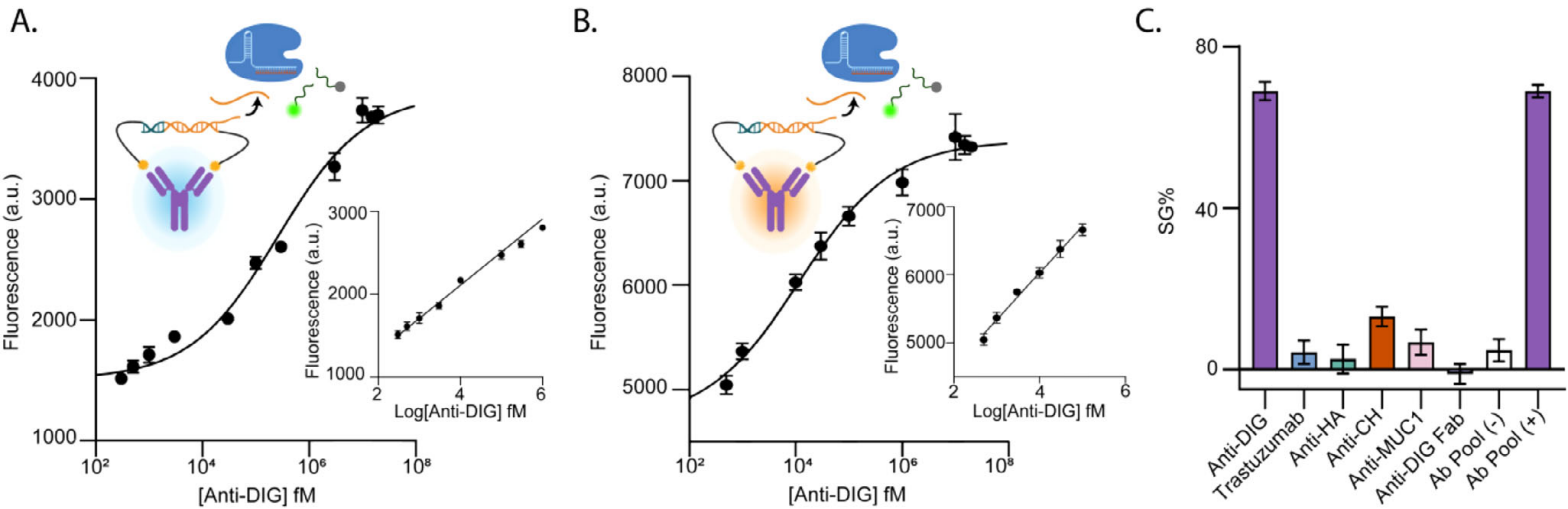
(A) Dose‐response curve showing an increase in fluorescence as a function of anti‐DIG antibody concentration in buffer. Inset: calibration curve obtained by linear fitting of endpoint fluorescence intensity values in the 300 fM—3 nM range (LOD = 230 fM). The curve is described by the following equation: Y = 400.1*X + 508.5, R^2^ = 0.975. (B) Dose‐response curve obtained in 10 % human serum supplemented with 5 mM Mg^2+^ (LOD=370 fM). Inset: calibration curve obtained by linear fitting of endpoint fluorescence intensity values in the 500 fM–100 pM range. The curve is described by the following equation: Y = 687.3*X + 3275, R^2^ = 0.975. (C) Specificity assessment of the sensing platform, comparing signal generated by anti‐DIG antibody (100 pM) vs. non‐target antibodies (100 pM), anti‐DIG Fab fragments (100 pM), and a pool of antibodies (trastuzumab, anti‐HA, anti‐CH, anti‐MUC1, 10 pM each) in the absence (‐) or presence (+) of anti‐DIG (100 pM). All experiments were conducted using 5 nM of nucleic acid components, 30 nM Cas13, and 100 nM RNA hairpin reporter at 25°C. The plotted intensity values were acquired after a 25 min cleavage reaction. Signal gain (%) was calculated relative to background fluorescence. The limit of detection (LOD) was calculated as the ratio of three times the standard deviation of the blank and the slope of the calibration curve. Error bars represent standard deviation across three independent replicates (n=3).

Following optimization, we evaluated the analytical performance of MARPLE using anti‐digoxigenin (anti‐DIG) antibody as a model antibody that recognizes a small molecule antigen. In this setup, a DIG‐DNA conjugate (DDC1) was used to sequester the RNA target strand required for Cas13 activation. Upon addition of the complementary DIG‐DNA conjugate (DDC2) and anti‐DIG antibodies, the antibody‐induced proximity effect triggered the release of the RNA activator. To quantify this response, we performed fluorescence kinetic measurements across a range of antibody concentrations from 300 fM to 30 nM. The nucleic acid components—including the DDC1–RNA heteroduplex, DDC2, and fluorescent RNA reporters—were first incubated with the antibody analyte for 15 min, after which Cas13 was added. Fluorescence was monitored for an additional 25 min, and endpoint values were used to generate calibration curves (Figure 3A; Figure S3), demonstrating femtomolar sensitivity with a limit of detection (LOD) of 230 fM. Fluorescence kinetics in the absence of target antibodies showed consistently low background levels (Figure S3), confirming that the measured signal gains reflect a robust signal‐to‐background separation.

Next, we tested MARPLE in diluted human serum from healthy male donors as a reference complex biological matrix. As human serum contains RNases that could degrade the CRISPR RNA target and the reporters, it was critical to investigate the integrity and functionality of our assay in these conditions. To assess this, we incubated these RNA components in increasing human serum concentration (up to 75 %) and monitored their stability over time. No degradation of either the hairpin structure or the ADC–Target heteroduplex was observed within 30 min based on the fluorescence signal remaining unchanged (Figure S4A,B), demonstrating that RNase activity does not pose constraints to our system under these conditions. To further minimize potential RNA degradation by RNases, we optimized the in‐serum protocol by first incubating Cas13, ADC1–Target, ADC2, and the antibody for 15 min before adding the RNA reporters. This configuration ensures that once the target is released, Cas13 can immediately recognize and cleave it, rapidly activating its trans‐cleavage activity against the RNA reporters. Introducing the reporters at this later stage minimizes their exposure to RNases and prevents nonspecific activation, thereby preserving a consistently high signal‐to‐background ratio (Figure S4C). Given the dependence on magnesium ions of both the toehold exchange reaction and Cas13 activity, we supplemented the serum samples with 5 mM Mg^2^⁺ to ensure optimal performance (Figure S4D). Under these conditions, MARPLE retained high sensitivity for anti‐DIG detection in 10 % serum, showing a LOD of 370 fM (Figure 3B; Figure S5). The assay also demonstrates high specificity, as a negligible fluorescence signal was observed in the presence of nonspecific compounds. Importantly, a pool of clinically relevant nonspecific antibodies in a complex matrix did not induce signal activation, whereas inclusion of anti‐DIG within the same pool restored the signal. Furthermore, using anti‐DIG Fab fragments failed to activate the system, confirming that RNA release is triggered by antibody‐mediated proximity requiring the full antibody structure (Figure 3C). To further demonstrate assay specificity, we tested MARPLE in the presence of random, non‐complementary RNA sequences that cannot activate Cas13. No signal gain was observed under these conditions, even in the presence of anti‐DIG, confirming the absence of off‐target Cas13 activity (Figure S6).

**FIGURE 4:**
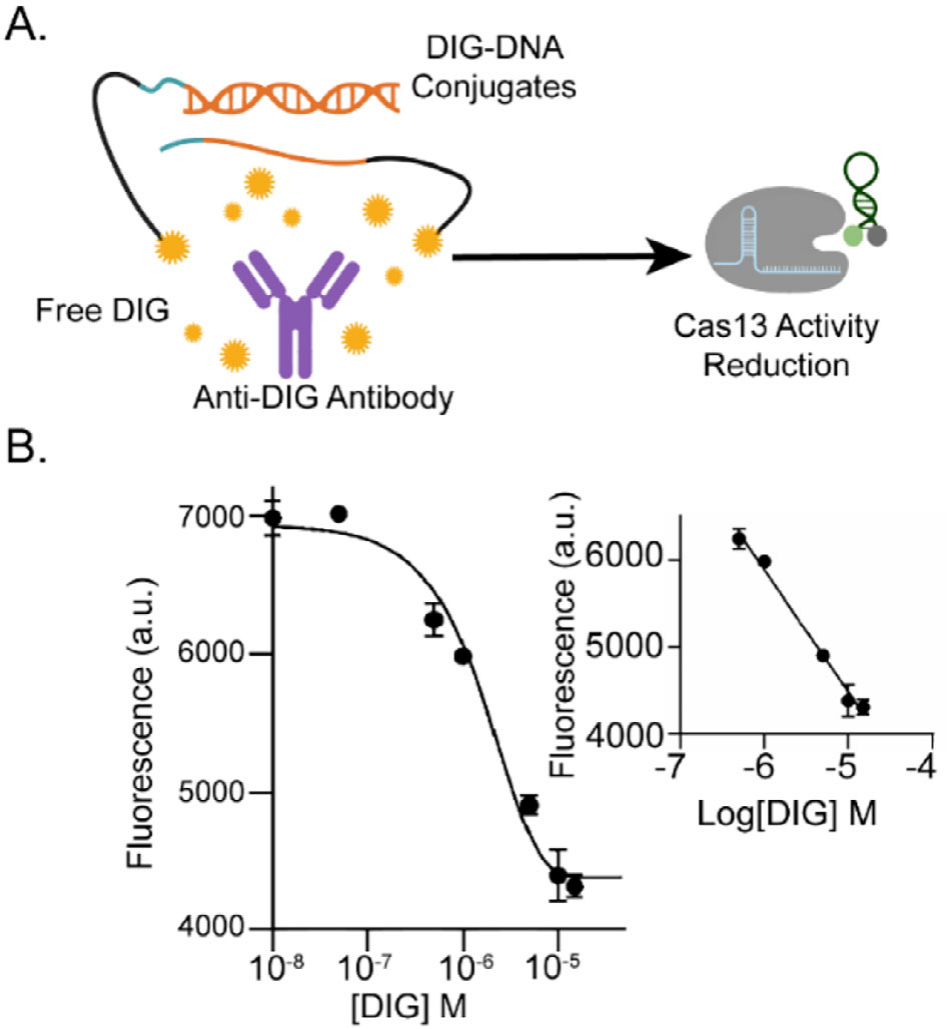
(A) Schematic representation of free digoxigenin detection using a competitive assay format. A fixed concentration of anti‐DIG antibody (1 nM) was incubated with equimolar nucleic acid components (5 nM each of DDC1–RNA target heteroduplex and DDC2) and Cas13 (30 nM). Increasing concentrations of free DIG were added prior to the introduction of the RNA hairpin reporter (100 nM). DIG competes with the DIG‐DNA conjugates for antibody binding, inhibiting RNA target release and reducing Cas13‐mediated signal generation. (B) Dose‐response curve showing decrease in fluorescence as a function of free DIG concentration in 10 % human serum supplemented with 5 mM Mg^2+^ (LOD = 89 nM). Inset: calibration curve obtained by linear fitting of endpoint fluorescence intensity values in the 0.5 µM—15 µM DIG concentration range. The curve is described by the following equation: Y = ‐1398*X ‐ 2501, R^2^ = 0.980. The limit of detection (LOD) was calculated as the ratio of three times the standard deviation of the blank and the slope of the calibration curve. Error bars represent standard deviation from three independent experiments (n=3).

To further address sample heterogeneity and assess robustness across varying biological matrix conditions, we challenged MARPLE with increasing human serum concentrations. The assay retained consistent performance across 20 %, 50 %, and up to 75 % serum (corresponding to undiluted serum after addition of MARPLE components; Figure S7), confirming that sensitivity and reliability are maintained even in highly complex biological matrices. In addition, MARPLE can be easily converted into a single‐step competitive assay for free antigen detection. Dose‐dependent inhibition of the proximity‐triggered reaction was obtained in the presence of free DIG, enabling quantitative detection of target free DIG in 10 % serum with LOD = 89 nM (Figure 4). To further demonstrate the versatility of the assay, we implemented a label‐free detection mode by replacing the RNA hairpin reporters with the fluorogenic RNA aptamer Mango, which folds into a specific secondary structure in the presence of KCl and binds the fluorophore thiazole orange (TO1) with high affinity, resulting in substantial fluorescence enhancement [49]. In this configuration, none of the assay components require covalent labeling, as the small‐molecule dye TO‐1 is simply added in solution and becomes fluorescent only upon binding the Mango aptamer, eliminating the need for costly custom‐labeled oligonucleotides.

Upon activation of Cas13 by the released RNA target, Mango RNA is cleaved, disrupting its structure and preventing fluorophore binding. This generates a signal‐OFF response modality, where higher concentrations of anti‐DIG led to greater Cas13 activity, increased Mango RNA degradation, and therefore decreased fluorescence (Figure S8). Finally, we demonstrated the generalization of MARPLE by developing an assay for a second small‐molecule‐binding antibody. Specifically, we adapted the assay for quantification of anti‐cholesterol (anti‐CH) antibodies by using cholesterol‐DNA conjugates, achieving detection of target antibodies with a LOD of 212 fM (Figure S9).

### 2.3 Modular Adaptation for Clinically Relevant Antibody Detection by Using PNA‐Peptide Conjugates

**FIGURE 5:**
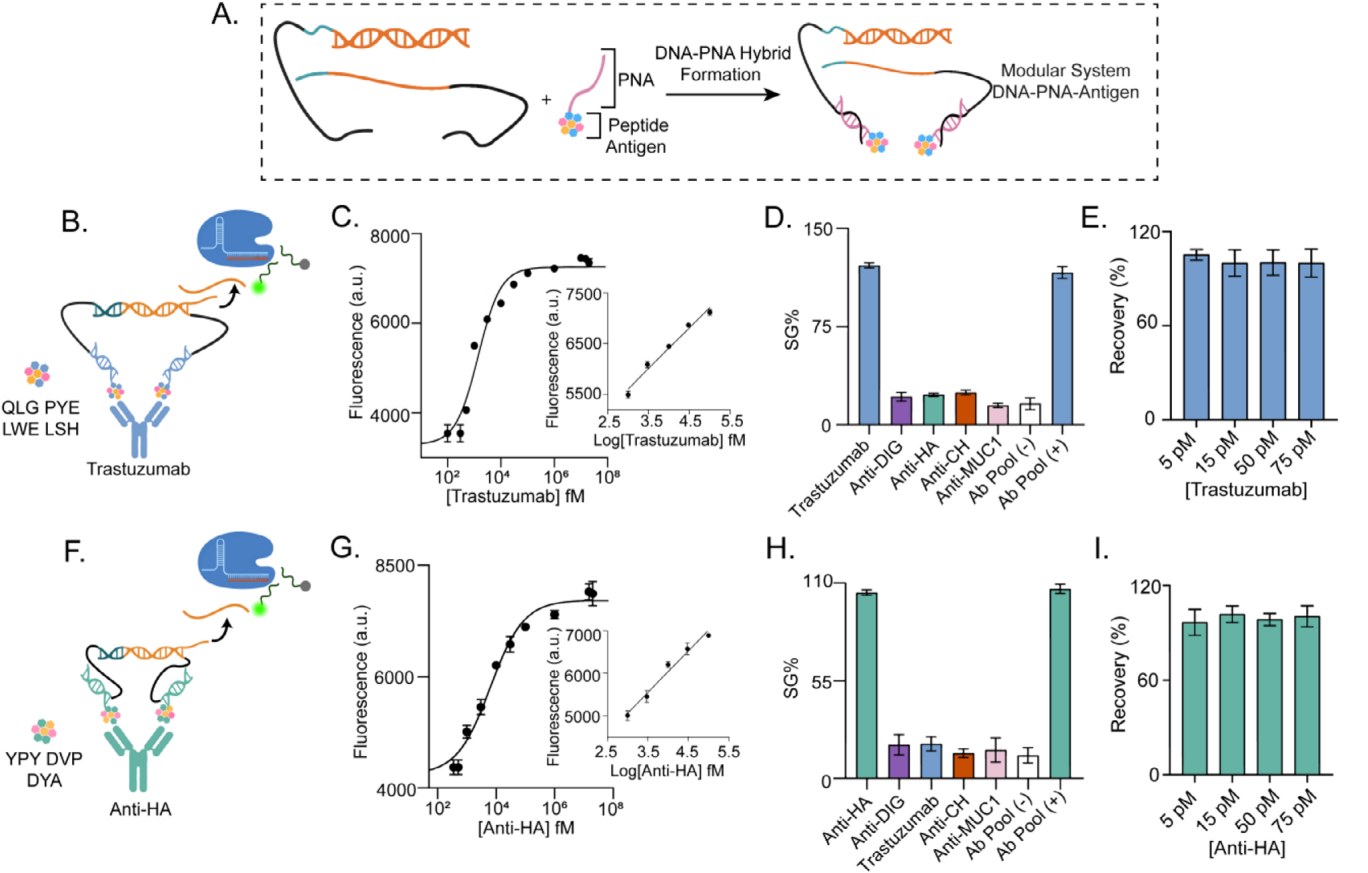
(A) Schematic representation of the modular MARPLE architecture using peptide–PNA conjugates as recognition elements hybridized to DNA handles. (B) Illustrative cartoon of the assay configuration developed for trastuzumab detection. (C) Dose‐response curve showing increase in fluorescence as a function of trastuzumab concentration in 10 % serum supplemented with 5 mM Mg^2+^ (LOD=330 fM). Inset: calibration curves obtained by linear fitting of endpoint fluorescence intensity values in the 500 fM–100 pM concentration range. The curve is described by the following equations: Y = 804.7*X + 3193, R^2^ = 0.972. (D) Specificity assessment of the sensing platform, comparing signal generated by trastuzumab (100 pM) vs. non‐target antibodies (100 pM) and pool of antibodies (anti‐DIG, anti‐HA, anti‐CH, anti‐MUC1, 10 pM each) in the absence (‐) or presence (+) of trastuzumab (100 pM). (E) Recovery tests were performed in 10 % serum spiked with varying concentrations of trastuzumab. (F) Illustrative cartoon of the assay configuration developed for anti‐HA detection. (G) Dose‐response curve showing increase in fluorescence as a function of anti‐HA concentration in 10 % serum supplemented with 5 mM Mg^2+^ (LOD=358 fM). Inset: calibration curves obtained by linear fitting of endpoint fluorescence intensity values in the 500 fM–100 pM concentration range. The curve is described by the following equations: Y = 977.9*X + 2127, R^2^ = 0.962. (H) Specificity assessment of the sensing platform, comparing signal generated by anti‐HA antibody (100 pM) vs. non‐target antibodies (100 pM) and pool of antibodies (anti‐DIG, trastuzumab, anti‐CH, anti‐MUC1, 10 pM each) in the absence (‐) or presence (+) of anti‐HA (100 pM) (I) Recovery tests performed in 10 % serum spiked with varying concentrations of anti‐HA. All experiments were conducted using 5 nM of nucleic acid components, 30 nM Cas13, and 100 nM RNA hairpin reporter at 25°C. The plotted intensity values were acquired after a 25 min cleavage reaction. Signal gain (%) was calculated relative to background fluorescence. Error bars indicate the standard deviation observed across three independent experiments (n=3).

By simply modifying the recognition elements conjugated to the oligonucleotides, MARPLE can be adapted for the detection of a broad range of target antibodies. To demonstrate this adaptability, we expanded the platform beyond small molecule–DNA conjugates (used in the detection of anti‐DIG and anti‐CH antibody detection) to a modular architecture capable of targeting clinically relevant antibodies. We first applied MARPLE to the detection of Trastuzumab, a therapeutic monoclonal antibody used in the treatment of HER2‐positive breast cancer. Trastuzumab detection plays a critical role in clinical oncology by supporting therapeutic monitoring, dose optimization, and toxicity minimization [50]. Accurate quantification enables clinicians to assess therapeutic levels and antibody clearance in patients undergoing HER2‐targeted therapy. In this new design, the oligonucleotide components no longer carry the antigen moiety directly. Instead, they are extended with a DNA handle that hybridizes to a PNA (peptide nucleic acid)‐peptide conjugate. The peptide, selected for specific binding to the target antibody, serves as the recognition element (Figure 5A,B,F).

This modular design circumvents the need for direct peptide–DNA conjugation—a step that is often costly and synthetically challenging—by enabling straightforward synthesis of PNA–peptide conjugates in a single round of solid‐phase peptide synthesis. Moreover, PNA–DNA hybridization decouples the recognition element from the core sensing strand, offering a flexible, scalable, and cost‐effective strategy for rapid adaptation to diverse antibody targets. In this design, the same DNA handles can be reused across assays, and only the peptide portion of the PNA–peptide module needs to be adapted for a new target antibody [51, 52]. For Trastuzumab detection, we introduced a PNA−peptide hybrid composed of a 17‐residue PNA domain conjugated to a 12‐aa‐long peptide (sequence: QLGPYELWELSH) recognized by Trastuzumab [38, 53]. Under the same reaction conditions as in the above assays, this modular version of MARPLE successfully detected Trastuzumab in 10 % human serum, with a LOD of 330 fM (Figure 5C; Figure S10). Notably, circulating trastuzumab levels after administration are typically in the µg/mL range. Given that the MARPLE LOD of 330 fM corresponds to concentrations in the pg/mL range, the assay comfortably covers the clinically relevant window. In practice, sample analysis would often require only simple dilution. The system demonstrated high specificity, with signal responses observed exclusively in the presence of the target antibody and no significant cross‐reactivity to nonspecific antibodies, even when these were present in the pool without trastuzumab. In contrast, when trastuzumab was included, signal gain was restored (Figure 5D). To benchmark MARPLE's performance against conventional methods, we carried out spike‐and‐recovery experiments in serum using known concentrations of trastuzumab (5, 15, 50, and 75 pM). Spike‐and‐recovery experiments involve spiking known amounts of antibody into serum and quantifying them with the assay; by comparing measured vs. expected concentrations, they provide a direct measure of assay accuracy in complex biological matrices. MARPLE yielded recovery rates between 90 % and 110 %, confirming its accuracy, reliability, and robustness (Figure 5E). Importantly, MARPLE demonstrated the same level of accuracy as an anti‐Trastuzumab ELISA, while offering the distinct advantages of one‐pot operation, a minimal‐workflow protocol, and superior sensitivity, making it especially attractive for rapid, user‐friendly immunodiagnostic applications (Figures S11 and S12). To further demonstrate the versatility of this modular format, we developed a MARPLE assay for the detection of anti‐hemagglutinin (anti‐HA) antibodies. Hemagglutinin is a surface protein of the influenza virus, and anti‐HA antibodies are key indicators of immune protection following infection or vaccination. Monitoring anti‐HA levels supports vaccine efficacy assessment, population immunity tracking, and evaluation of individual immune responses. A rapid, sensitive method for anti‐HA detection thus has broad utility in infectious disease diagnostics and immunological research [54]. For this adaptation, we used a recognition element consisting of 9‐PNA residues conjugated with a 9‐aa peptide epitope derived from the HA protein, which had already been used and validated in previous work [42]. By retaining the same RNA components used in previous setups, thereby reducing both assay complexity and the cost associated with the Cas13 system, we accommodated this PNA–peptide construct into MARPLE's architecture (Figure 5F). The assay successfully detected anti‐HA antibodies in 10 % human serum with a LOD of 358 fM (Figure 5G; Figure S13). Here too, clinical anti‐HA serum levels are typically in the µg/mL range, while the MARPLE LOD falls in the pg/mL range, indicating that only simple sample dilution would be required for practical use. As with the Trastuzumab assay, the system showed high specificity (Figure 5H) and recovery rates between 90 % and 110 % (Figure 5I), matching the same level of accuracy of an in‐house anti‐HA ELISA (Figure S14).

### 2.4 Anti‐MUC1 Antibody Detection by Using Whole Protein‐DNA Conjugates

While the modular MARPLE format using peptide‐PNA conjugates as recognition elements offers clear advantages—such as design flexibility, synthetic accessibility, and controlled specificity—using peptide fragments as antigens may pose certain limitations in complex biological samples. Naturally occurring antibodies are typically polyclonal and can recognize a broad array of epitopes on the same target, including conformational and discontinuous sites present only in full‐length proteins. As a result, relying solely on peptide fragments may fail to detect antibodies that recognize other regions of the antigen, thereby limiting the system's overall sensitivity and effectiveness.

**FIGURE 6:**
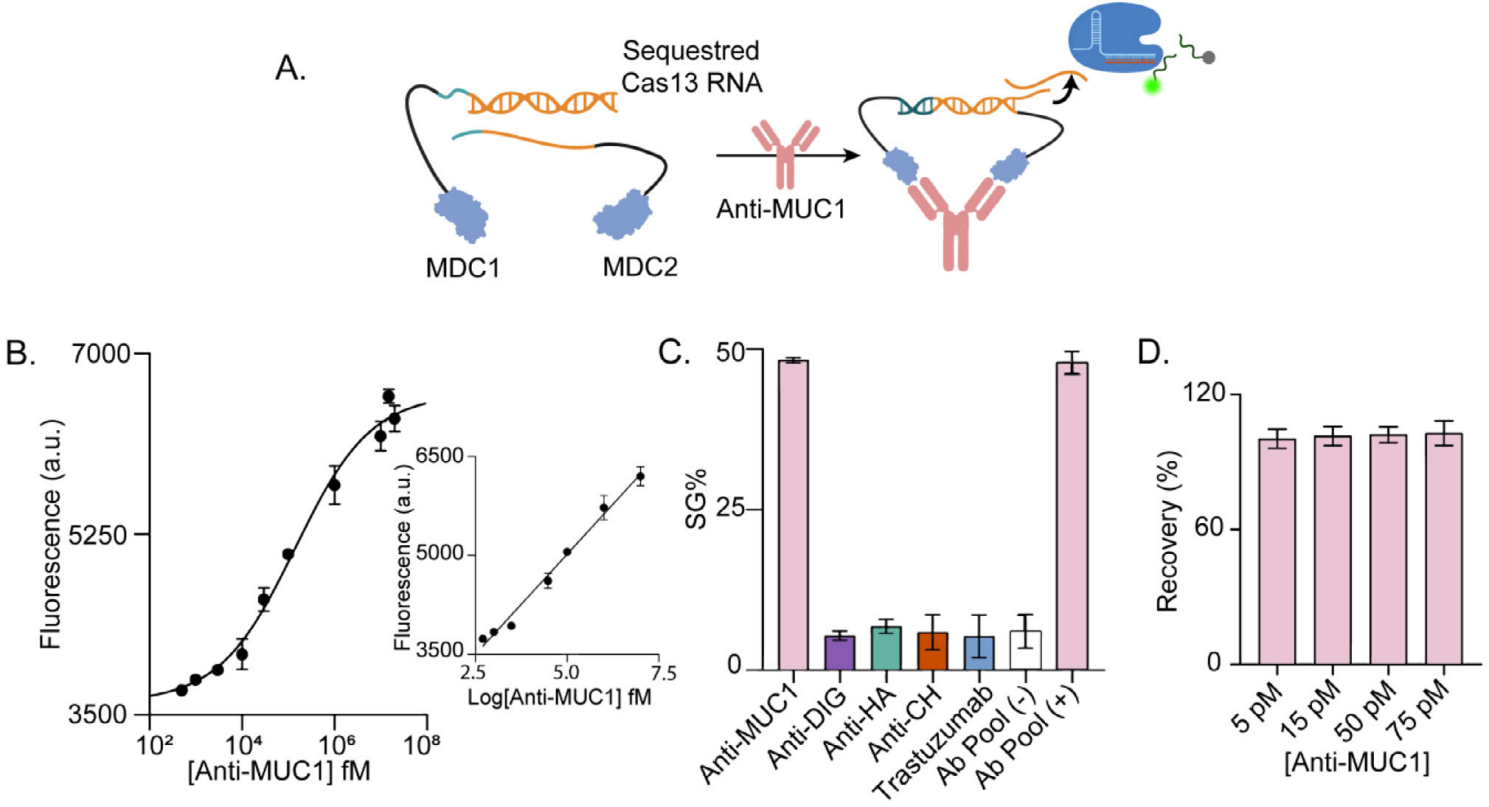
(A) Illustrative cartoon of the assay configuration developed for anti‐MUC1 detection using MUC1–DNA conjugates. (B) Dose‐response curve showing increase in fluorescence as a function of Anti‐MUC1 concentration in 10 % serum supplemented with 5 mM Mg^2+^ (LOD=261 fM). Inset: calibration curves obtained by linear fitting of endpoint fluorescence intensity values in the 500 fM—10 nM concentration range. The curve is described by the following equations: Y = 633.2*X + 1889, R^2^ = 0.983. (C) Specificity assessment of the sensing platform, comparing signal generated by anti‐MUC1 antibody (100 pM) vs. non‐target antibodies (100 pM) and pool of antibodies (anti‐DIG, anti‐HA, anti‐CH, trastuzumab, 10 pM each) in the absence (‐) or presence (+) of anti‐MUC1 (100 pM). (D) Recovery tests in 10 % serum spiked with varying concentrations of anti‐MUC1. All experiments were conducted using 5 nM of nucleic acid components, 30 nM Cas13, and 100 nM RNA hairpin reporter at 25°C. The plotted intensity values were acquired after a 25 min cleavage reaction. Signal gain (%) was calculated relative to background fluorescence. Error bars indicate the standard deviation observed across three independent experiments (n=3).

To address this challenge, we explored using whole protein‐DNA conjugates as the components of the assay. We developed a new version of MARPLE in which ADC1 and ADC2 are directly conjugated to the full extracellular domain of mucin 1 (MUC1), a clinically relevant tumor‐associated antigen. Each MUC1–DNA conjugate (MDC1 and MDC2) was synthesized by covalently attaching the relevant DNA strand to lysine residues in the extracellular domain of MUC1 protein using the proFIRE platform (Figure 6A,B; Figure S15). These constructs are intended for the detection of anti‐MUC1 autoantibodies—a class of cancer‐associated antibodies produced in response to abnormal MUC1 expression. The presence of such antibodies in patient serum has been associated with disease stage, prognosis, and immune status, establishing their relevance as both diagnostic and prognostic biomarkers, as well as indicators for patient stratification in MUC1‐targeted immunotherapy and vaccine strategies [55, 56, 57, 58]. After MDC1 and MDC2 synthesis (Figure S15), we evaluated this version of MARPLE in 10 % human serum, achieving a LOD of 261 fM for anti‐MUC1 antibodies (Figure 6C; Figure S16). Clinical anti‐MUC1 levels are typically reported in relative units (arbitrary units, or assay‐specific thresholds), which prevents direct cross‐patient standardization and makes detectability highly dependent on assay sensitivity. MARPLE's femtomolar detection limit lies well below reported values, highlighting its potential as a highly sensitive and broadly applicable platform for anti‐MUC1 detection. The assay showed high specificity, generating strong signal responses exclusively in the presence of the target antibody, with no significant cross‐reactivity to unrelated antibodies (Figure 6D). To benchmark its performance, we conducted spike‐and‐recovery experiments in serum using known concentrations of anti‐MUC1 (5, 15, 50, and 75 pM). MARPLE demonstrated excellent accuracy, with recovery rates ranging from 90 % to 110 %, comparable to those obtained with an in‐house anti‐MUC1 ELISA (Figure S17), confirming its reliability and robustness in complex biological matrices (Figure 6E). This approach of incorporating the full‐length antigen aims to maximize the diversity and functionality of antigen–antibody interactions within the assay. This is particularly relevant for polyclonal antibodies that can recognize multiple epitopes across the protein surface. However, for assays designed to detect monoclonal antibodies, site‐specific protein‐DNA conjugation may be required to ensure efficient and reproducible recognition, as the location of conjugation sites relative to the antibody binding epitope can have a decisive impact on the proximity mechanism and assay performance.

## 3 Conclusions

In this work, we introduced MARPLE (Modular Antibody Recognition via Proximity‐triggered Linker Exchange), a fully modular, one‐pot CRISPR‐Cas13–based immunoassay for ultrasensitive and specific antibody detection. We demonstrated that MARPLE achieved consistent femtomolar sensitivity and high specificity across a diverse panel of antibody targets, including small molecule–binding antibodies (anti‐digoxigenin, anti‐cholesterol), therapeutic antibodies (trastuzumab), pathogen‐specific antibodies (anti‐HA), and cancer‐associated autoantibodies (anti‐MUC1). Importantly, the assay retained robust performance in complex biological matrices such as human serum. Through three modular formats—direct antigen–DNA conjugation, peptide–PNA hybridization, and whole protein–DNA conjugates—MARPLE offers flexible and scalable adaptation to clinically relevant antibodies without compromising analytical performance. Compared to Cas12‐based proximity assays, MARPLE employs a fundamentally different activation strategy. Instead of relying on proximity‐triggered enzymatic generation of a new DNA substrate for Cas12, MARPLE uses a proximity‐gated toehold‐exchange reaction that liberates an RNA activator under conditions in which the reaction would otherwise be disfavored. In this framework, the antibody functions as a driver of effective molarity, enabling a DNA/RNA strand‐exchange process that does not occur in the absence of the target. Compared to previous CRISPR Cas13‐based immunoassays such as MAIGRET, MARPLE operates in a straightforward, one‐pot reaction format, eliminating the need for in vitro transcription steps. This results in faster analysis within 30 min, simplified workflows, and detection limits in the femtomolar range, providing enhanced sensitivity compared to conventional immunoassays (Figure S12). These features highlight MARPLE as a versatile and broadly applicable tool for immunodiagnostics, expanding the scope of CRISPR‐based sensing to antibody biomarkers.

## 4 Experimental Section

### 4.1 Oligonucleotides

The DNA sequences were designed and analyzed using the NUPACK software (www.nupack.org) [59, 60, 61] and were purchased from Metabion International AG (Planegg, Germany). All oligonucleotides were dissolved in water at a concentration of 100 µM and stored at −20°C.

### 4.2 Human Serum

The human serum used in this work is commercially available human serum originating exclusively from healthy male donors of blood group AB, supplied by Capricorn Scientific (Cat. No. HUM‐3B; Ebsdorfergrund, FRA, Germany).

### 4.3 Formation of the Cas13‐crRNA Complex

To form the Cas13‐crRNA complex, a solution of Cas13 and crRNA, 1 µM for both components, was prepared in PBS containing 5 mM Mg^2+^. The solution was placed in a dry block thermostat set to 37°C for a 30 min incubation period.

### 4.4 Fluorescence Measurements in Buffer

Annealing of ADC1 and the RNA target was performed at a final concentration of 5 nM each. ADC2 was then added to the mixture at a final concentration of 5 nM, together with varying concentrations of antibody. The solution was incubated for 15 min at 25°C, together with RNA hairpin reporter (100 nM). Subsequently, CRISPR‐Cas13 complex (30 nM) was added to initiate the trans‐cleavage reaction. The final reaction volume was 110 µL. Spectral acquisition was conducted using an excitation wavelength (λ_exc_) of 538 nm and an emission range (λ_em_) of 555–595 nm. Fluorescence measurements were performed at λ_exc_ = 538 nm and λ_em_ = 564 nm over a total duration of 30 min, with the trans‐cleavage reaction monitored for 25 min. All measurements were conducted at 25°C. Kinetic acquisitions were carried out using a Fluoromax‐3 (150 W continuous Xe source; excitation monochromator 200‐950 nm; emission monochromator 200‐950 nm; “Photon counting” emission detector with photomultiplier optimized for 290‐850 nm; automatic Glan Thompson polarizers; cuvette: Ultra‐Micro Cell 10 × 2 mm^2^, volume 100 µL, center height 15 mm, purchased from Hellma Analytics).

### 4.5 Fluorescence Measurements in Serum

Annealing of ADC1 and the RNA target was performed at a final concentration of 5 nM each. ADC2 was then added to the mixture at a final concentration of 5 nM, together with 10 %, 20 %, 50 % or 75 % human serum and varying concentrations of antibody. The solution was incubated for 15 min at 25°C together with CRISPR‐Cas13 complex (30 nM). Subsequently, RNA hairpin reporter (100 nM) was added to initiate the trans‐cleavage reaction. The final reaction volume was 110 µL. Spectral acquisition was conducted using an excitation wavelength (λ_exc_) of 538 nm and an emission range (λ_em_) of 555–595 nm. Fluorescence measurements were performed at λ_exc_ = 538 nm and λ_em_ = 564 nm over a total duration of 30 min, with the trans‐cleavage reaction monitored for 25 min. All measurements were conducted at 25°C. Kinetic acquisitions were carried out using a Fluoromax‐3 (150 W continuous Xe source; excitation monochromator 200–950 nm; emission monochromator 200–950 nm; “Photon counting” emission detector with photomultiplier optimized for 290–850 nm; automatic Glan Thompson polarizers; cuvette: Ultra‐Micro Cell 10 × 2 mm^2^, volume 100 µL, center height 15 mm, purchased from Hellma Analytics).

### 4.6 Specificity Tests

Specificity tests were conducted under the same conditions described according to fluorescent measurement in serum protocol, using non‐target antibodies (10 nM) instead of the specific one associated with the DNA system. Signal gain (%) is calculated after 25 min of trans cleavage activity, according to the formula:

$$Signal\;Gain\;\%=\frac{Fluorescence\;Signal-Background}{Background}x100$$

where *Fluorescence Signal* refers to the fluorescence intensity measured in the presence of the antibody, while *Background* corresponds to the signal observed in the absence of the antibody.

### 4.7 Spike‐and‐Recovery Tests

Spike‐and‐recovery experiments were performed in 10 % human serum. Known concentrations of the target antibody, chosen within the linear range of the calibration curve, were spiked into serum samples and subsequently analyzed with the MARPLE assay. Based on the resulting fluorescence intensities, antibody concentrations were interpolated from the linear calibration equation and compared with the nominal spiked values. Recovery rates were calculated according to the following equation:

$$Recovery\left(\%\right)=\frac{Measured\;Concentration}{Spiked\;Concentration}x100$$

where *Measured Concentration* refers to the value interpolated from the linear calibration curve, and *Spiked Concentration* corresponds to the nominal antibody concentration added to the sample.

### 4.8 ELISA

Enzyme‐linked immunosandwich assay (ELISA) as a reference technique for the detection of anti‐HA and anti‐MUC1 antibodies was performed following an in‐house standard ELISA protocol for colorimetric detection [62]. Detailed protocols can be found in the Supporting Information.

### 4.9 Data and Statistical Analysis

The concentration‐dependent assays were conducted to measure the signal variations in response to different antibody concentrations. End‐point intensity values vs. concentration of antibody were fitted with the following four‐parameter logistic equation:

$$\begin{array}{ccc} Fluorescence\; & Intensity & \left[Ab\right]\\ & & =B_{min}+\left(B_{max}-B_{min}\right)\frac{{\left[Ab\right]}^{n_{H}}}{K_{\frac{1}{2}}^{n_{H}}+{\left[Ab\right]}^{n_{H}}} \end{array}$$

where [Ab] is the antibody concentration, B_min_ represents the background fluorescence in the absence of antibody, and B_max_ corresponds to the maximum fluorescence at saturation. $K_{\frac{1}{2}}$ denotes the antibody concentration at half‐maximum response, and n_H_ is the Hill coefficient. The limit of detection (LOD) was calculated based on the ratio of three times the standard deviation of the blank and the slope of the calibration curve. All datasets were pre‐processed by checking for outliers and by verifying the consistency of fluorescence baselines across independent replicates. Quantitative results are reported as mean ± standard deviation. For each experiment, the sample size (n) corresponds to three independent replicates (n = 3). All statistical analyses and curve fittings were performed using GraphPad Prism (version 8).

## Conflicts of Interest

The authors declare no conflicts of interest.

## Supporting information




**Supporting file**: advs73447‐sup‐0001‐SuppMat.docx.

## Data Availability

The data that support the findings of this study are available from the corresponding author upon reasonable request.
