# Supplementary material for: MARPLE: A Proximity‐Triggered CRISPR‐Cas13 Platform for Ultrasensitive Antibody Detection
Source: Adv Sci (Weinh). 2025 Dec 22;13(13):e17799. doi: 10.1002/advs.202517799 (PMC12955873; doi:10.1002/advs.202517799)
Supplement: Supplementary file 1 — Supporting file: advs73447‐sup‐0001‐SuppMat.docx. [file ADVS-13-e17799-s001.docx]

**Supplementary Information**

**MARPLE: A Proximity-Triggered CRISPR-Cas13 Platform for Ultrasensitive Antibody Detection**

*Elena Spezzani,^a^ Luca Capelli,^a^ Denise di Lena,^a,c^ Alejandro Chamorro-Garcia,^b^ Rudy Ippodrino,^c^ Alessandro Porchetta ^b,d^ and Alessandro Bertucci* ^a,d^*

[a]E. Spezzani, L. Capelli, D. Di Lena, A. Bertucci

Department of Chemistry, Life Sciences and Environmental Sustainability

University of Parma

Parco Area delle Scienze, 17/A, 43124 Parma, Italy

E-mail: [alessandro.bertucci@unipr.it](mailto:alessandro.bertucci@unipr.it)

[b]A. Chamorro-Garcia, A. Porchetta

Department of Sciences and Chemical Technologies

University of Rome, Tor Vergata

Via della Ricerca Scientifica 1, 00133, Rome, Italy

[c] D. Di Lena, R. Ippodrino

Ulisse BioMed Laboratories, Area Science Park, 34149 Trieste, Italy

[d] A. Porchetta, A. Bertucci

Biostructures and Biosystems National Institute (INBB), Via dei Carpegna 19, 00165 Rome, Italy

**Table Of Contents**

[**Supplementary Experimental Procedures** 3](#_Toc216185387)

[**Reagents and Materials** 3](#_Toc216185388)

[**DNA System** 3](#_Toc216185389)

[**Native 10% Polyacrylamide Gel (PAA Gel)** 4](#_Toc216185390)

[**RNA Mango System** 4](#_Toc216185391)

[**Oligonucleotide-MUC1 conjugation** 4](#_Toc216185392)

[**Trastuzumab Detection by using ELISA** 4](#_Toc216185393)

[**Anti-HA Detection by using ELISA** 5](#_Toc216185394)

[**Anti-MUC1 Detection by using ELISA** 6](#_Toc216185395)

[**DNA Sequences** 6](#_Toc216185396)

[**Supplementary Figures** 11](#_Toc216185397)

[**Supplementary References** 28](#_Toc216185398)

## **Supplementary Experimental Procedures**

## **Reagents and Materials**

PBS buffer solution was purchased from Corning (Manassas, VA, USA). RNAse Free water was supplied by IDT. TO1-3PEG-Biotin was purchased from abm (Applied Biological Materials, Richmond, BC, Canada). Tween 20, bovine serum albumin (BSA), sodium carbonate (Na2CO3), sodium bicarbonate (NaHCO3), sodium hydroxide (NaOH), sodium dihydrogen phosphate (NaH2PO4), disodium hydrogen phosphate (Na2HPO4), sulfuric acid (H2SO4), potassium chloride (KCl2), MgCl_2,_ 3,3′,5,5′-Tetramethylbenzidine (TMB) reagent for ELISA, Anti-DIG and anti-HA antibodies were purchased from Sigma Aldrich (St. Louis, MO). Human serum, type AB (male) was purchased by Capricorn Scientific (Ebsdorfergrund, FRA, Germany). LwCas13a was purchased from Signalchem Diagnostics (Richmond, BC, Canada), anti-CH antibody by MyBiosource (San Diego, CA, USA) and Trastuzumab by Evidentic GmbH (Berlin, Germany). MUC1 and anti-MUC1 were purchased from SinoBiological (Beijing, China). PNA-Peptide chimera probes were purchased from Panagene (South Korea). Trastuzumab ELISA kit – Free drug was purchased from Assay Gene (Dublin, Ireland). Hemagglutinin epitope peptide (HA Tag3x) was purchased from AnaSpec Inc (Freemont, CA, US), rat monoclonal Anti-HA antibody was purchased from Roche (Mannheim, Germany), Goat anti mouse IgG labelled with HRP (anti mouse IgG-HRP) was purchased from Biorbyt (Cambridge, UK). Mouse monoclonal Anti-MUC1 was purchased from Merck (Milan, Italy). Multiwell plates F96 maxisorp NUNC-immunolpate was purchased from thermofisher (Roskilde, Denmark).

**DNA System**

Annealing of the DNA-based antibody mimic was performed in PBS supplemented with 5 mM Mg²⁺. Ab_M1 and Ab_M2 were mixed at a final equimolar concentration of 500 nM. Similarly, annealing was carried out between s1_th(n) and the labeled DNA target mimic under the same conditions. s1_th(n)-DNA target mimic homodouplex and the respective s2_th(n) were added in the same solution at a final concentration of 150 nM. DNA-based antibody mimic was then added to a final concentration of 150 nM. The toehold exchange reaction was monitored with kinetic measurement, at λ_exc_ = 488 nm and λ_em_= 520 nm over a total duration of 60 minutes. All measurements were conducted at 25 °C.

Kinetic acquisitions were carried out using a Fluoromax-3 (150W continuous Xe source; excitation monochromator 200-950 nm; emission monochromator 200-950 nm; "Photon counting" emission detector with photomultiplier optimized for 290-850 nm; automatic Glan Thompson polarizers; cuvette: Ultra-Micro Cell 10x2 mm, volume 100 μl, center height 15 mm, purchased from Hellma Analytics).

**Native 10% Polyacrylamide Gel (PAA Gel)**

A 10% native polyacrylamide gel was prepared by diluting a 40% acrylamide solution in 1X Tris-Borate EDTA (TBE) buffer in a 15 mL Falcon tube. Polymerization was initiated by adding 10% ammonium persulfate (APS, dissolved in milliQ water) and TEMED. The mixture was poured between glass plates, a comb was inserted, and the gel was allowed to polymerize. Samples containing 5 nM nucleic acid components and 10 nM anti-DIG antibody were loaded alongside GeneRuler Ultra Low Range DNA Ladder (Thermo Scientific). The gel was run in 1X TBE at 140 mV for 40 minutes. Following the run, the gel was stained with Syber Gold for 30 minutes in the dark with slow oscillation.

**RNA Mango System**

Annealing of ADC1 and the RNA target was performed at a final concentration of 5 nM each. ADC2 was then added to the mixture at a final concentration of 5 nM, together with varying concentrations of anti-DIG antibody. The solution was incubated for 15 minutes at 25 °C. Subsequently, Mango RNA (100 nM) and CRISPR-Cas13 complex (30 nM) were added to initiate the trans-cleavage reaction. The final reaction volume was 110 μL. The trans cleavage reaction was let occur for 25 minutes, after which 4 M KCl and 65 μM TO-1 were added, and a fluorescence spectrum was subsequently recorded (λexc= 505 nm and λem= 515-580 nm) on a FP-8259 Jasco Inc (Hachioji, Tokyo, Japan) fluorometer.

**Oligonucleotide-MUC1 conjugation**

To obtain MDC1 and MDC2, the amine-coupling kit provided by Dynamic Biosensors GmbH was used. Following the manufacturer’s instructions, oligonucleotides functionalized with DBCO at the 5′ end were first activated exploiting a crosslinker. The activated oligonucleotides (s1 and s2) were then added to separate solutions containing 100 µg of protein each. Conjugation with the protein’s amine groups was carried out at room temperature for 1 hour, followed by overnight incubation at 4 °C. The resulting conjugates were purified using the proFIRE system (Dynamic Biosensors GmbH) and subsequently subjected to buffer exchange into PBS supplemented with 5 mM Mg²⁺. Only conjugates with stoichiometric equality—one DNA oligonucleotide per protein—were collected. Quantification was performed using a NanoDrop 1000 spectrophotometer (Thermo Fisher Scientific, Monza, Italy) by measuring absorbance at 260 nm.

**Trastuzumab Detection by using ELISA**

Enzyme-linked immunosandwich assay (ELISA) as reference technique for the detection of Trastuzumab was performed following a standard ELISA protocol for colorimetric detection of a commercial Kit. Commercial multiwell plates already modified with a capture anti-Trastuzumab antibodies and blocked with BSA to prevent nonspecific interactions. Briefly, standards were diluted at working concentrations either in assay buffer (samples in buffer) or 10% serum in assay buffer (for the 10% Serum samples), 100 µL were added into the corresponding wells in the commercial plate and incubated for 1h at room temperature (around 25 °C). Next, 3 washing cycles were performed, all washings unless otherwise stated consisted in cycles of emptying the well content and adding 200 µL per well of washing buffer: 0.05 % (v/v) Tween 20 in pH 7.4 Phosphate buffered saline (PBS). Next, we added 100 µL of conjugate (anti human -HRP) antibody to each well and incubated for 1h at room temperature. Last, 3 standard washings cycles and one final washing cycle in just PBS were performed, then, 100 µL of colorimetric substrate for HRP were added into each well. The reaction was stopped after 2 min by adding 100 µL of 0.5 M H2SO4 in each well. Absorbance signal was read at 450 nm using Tecan Infinite M200 PRO (Männedorf, Switzerland).

**Anti-HA Detection by using ELISA**

Enzyme-linked immunosandwich assay (ELISA) as reference technique for the detection of anti-HA antibody was performed following an in-house standard ELISA protocol for colorimetric detection.^[62]^ Briefly, maxisorp® multiwell plates were coated by adding 100 µL per well of the HA peptide (HA) at 1 µg/mL, in 50 mM carbonate buffer pH 9.0, as capturing recognition element, and incubated at 4 °C overnight. The next day the plates were washed 3 times; all washing steps unless otherwise stated consisted in cycles of emptying the content in the wells and adding 200 µL per well of washing buffer: 0.05 % (v/v) Tween 20 in Phosphate buffered saline (PBS). Next, to prevent nonspecific interactions 200 µL of blocking solution, 2% (w/v) BSA in PBS, were added to each well, and incubated for 2h at 37 °C. Then, 3 washing cycles were performed, and 100 µL of sample containing the target (anti-HA antibodies) in its’ corresponding conditions, either incubation buffer or 10% (v/v) serum in incubation buffer, were added to its corresponding well and incubated for 1h at 37 °C (unless otherwise stated incubation buffer composition was : 1% (w/v) BSA 0.05% (v/v) Tween 20 pH 7.4 PBS). Following, 3 washing cycles were performed, and 100 µL of goat anti mouse IgG labelled with horseradish peroxidase (anti mouse IgG-HRP) at 2 µg/ml in incubation buffer were added to each well and incubated for 1h at 37 °C. Last, 3 standard washing cycles and one final washing cycle in just PBS were performed, then 100 µL of HRP substrate commercial mix (TMB) were added to each well. Reaction was stopped after 0.5 minutes by adding 100 µL of 0.5 M H_2_SO_4_ per well. Absorbance signal was read at 450 nm using Tecan Infinite M200 PRO (Männedorf, Switzerland).

**Anti-MUC1 Detection by using ELISA**

Enzyme-linked immunosandwich assay (ELISA) as reference technique for the detection of anti-MUC antibody was performed following an in-house standard ELISA protocol for colorimetric detection.^[62]^ Briefly, maxisorp® multiwell plates were coated by adding 100 µL per well of a peptide-PNA chimera probe at 1 µg/mL, in 50 mM carbonate buffer pH 9.0, as capturing recognition element, and incubated at 4 °C overnight. The next day the plates were washed 3 times; all washing steps unless otherwise stated consisted in cycles of emptying the content in the wells and adding 200 µL per well of washing buffer: 0.05 % (v/v) Tween 20 in Phosphate buffered saline (PBS). Next, to prevent nonspecific interactions 200 µL of blocking solution, 2% (w/v) BSA in PBS, were added to each well, and incubated for 2h at 37 °C. Then, 3 washing cycles were performed, and 100 µL of sample containing the target (anti-MUC antibodies) in its’ corresponding conditions, either incubation buffer or 10% (v/v) serum in incubation buffer, were added to its corresponding well and incubated for 1h at 37 °C (unless otherwise stated incubation buffer composition was : 1% (w/v) BSA 0.05% (v/v) Tween 20 pH 7.4 PBS). Following, 3 washing cycles were performed, and 100 µL of goat anti mouse IgG labelled with horseradish peroxidase (anti mouse IgG-HRP) at 1 µg/ml in incubation buffer were added to each well and incubated for 1h at 37 °C. Last, 3 standard washing cycles and one final washing cycle in just PBS were performed, then 100 µL of HRP substrate commercial mix (TMB) were added to each well. Reaction was stopped after 1 minutes by adding 100 µL of 0.5 M H2SO4 per well. Absorbance signal was read at 450 nm using using Tecan Infinite M200 PRO (Männedorf, Switzerland).

**DNA Sequences**

**Table S1. DNA System**

| **Name** | **Sequence (5’- 3’)** | Figure |
| --- | --- | --- |
| Antibody Mimic1 (Ab_M1) | CGTGAACTAGGTATGCAGAGCTTCGATGC | Supplementary Figure S1 |
| Antibody Mimic2 (Ab_M2) | GATCGGTAACTGAGGCATCGAAGCTCTGC | Supplementary Figure S1 |
| DNA Target Mimic | *(6-FAM)- TAGCTTATCAGACTGATGTTGA* | Supplementary Figure S1 |
| s1_th2 | *CTCAGTTACCGATCTCTCCA****AG****TCAACATCAGTCTGATAAGCTA-(BHQ1)* | Supplementary Figure S1 |
| s2_th2 | *ATACCTAGTTCACG TCTCCAATCAGACTGATGTTGA****CT*** | Supplementary Figure S1 |
| s1_th3 | *CTCAGTTACCGATCTCTCCA****CAG****TCAACATCAGTCTGATAAGCTA-(BHQ1)* | Supplementary Figure S1 |
| s2_th3 | *ATACCTAGTTCACGTCTCCAATCAGACTGATGTTGA****CTG*** | Supplementary Figure S1 |
| s1_th4 | *CTCAGTTACCGATCTCTCCA****ACAG****TCAACATCAGTCTGATAAGCTA-(BHQ1)* | Supplementary Figure S1 |
| s2_th4 | *ATACCTAGTTCACGTCTCCAATCAGACTGATGTTGA****CTGT*** | Supplementary Figure S1 |
| s1_th5 | *CTCAGTTACCGATCTCTCCA****TACAG****TCAACATCAGTCTGATAAGCTA-(BHQ1)* | Supplementary Figure S1 |
| s2_th5 | *ATACCTAGTTCACGTCTCCAATCAGACTGATGTTGA****CTGTA*** | Supplementary Figure S1 |
| s1_th6 | *CTCAGTTACCGATCTCTCCA****CTACAG****TCAACATCAGTCTGATAAGCTA-(BHQ1)* | Supplementary Figure S1 |
| s2_th6 | *ATACCTAGTTCACGTCTCCAATCAGACTGATGTTGA****CTGTAG*** | Supplementary Figure S1 |

Here DNA sequences used in the preliminary experiments involving the DNA-based system (Supporting Figure 1). Ab Mimic strands 1 and 2 have the DNA-based epitope mimic underlined. The strands involved in the toehold exchange reaction (s1 and s2) have toehold portion (th) in bold and the DNA-based antigen mimic portion underlined.

**Table S2. Cas13 System RNA Sequences**

| **Name** | **Sequence (5’- 3’)** | **Figure** |
| --- | --- | --- |
| crRNA Cas13^[20,22,43]^ | ***GGGAUUUAGACUACCCCAAAAACGAAGGGGACUAAAA****CUCAACAUCAGUCUGAUAAGCUA* | 1-6, Supplementary Figures S3, S4C, S4D, S5-S7-S10, S13, S16 |
| RNA Target | *UAGCUUAUCAGACUGAUGUUGA* | 1-6, Supplementary Figures S3, S4C, S4D, S5-S7-S10, S13, S16 |
| Hairpin RNA Reporter^[20,43,61]^ | *(Cy3)-CUCUCAUUUUAUAUUUAGAGAG-(BHQ2)* | 1-6, Supplementary Figures S3, S4C, S4D, S5-S7-S10, S13, S16 |
| Random RNA 1 | *UAAUUUCUACUAAGUGUAGAUCUGUG* | Figure S6 |
| Random RNA 2 | *UGAGGUAGUAGGUUGUAUAGUU* | Figure S6 |
| Random RNA 3 | *UAAUUUCUACUAAGUGUAGAUAUUCUGUGAAAUAAAGGUAA* | Figure S6 |

The crRNA sequence is depicted with the stem region in bold, which is recognized and bound by the LwaCas13a enzyme ^[22,43]^ and in underline the spacer portion, complementary to the RNA target. The RNA target has been designed with a moderate GC content (~36.4%), which minimizes the formation of secondary structures that might otherwise hinder recognition by the Cas13–crRNA complex once the target RNA is released. In the hairpin RNA reporter sequence, the *underlined* segments denote the stem-forming regions, while the loop contains a poly-U/A sequence that is the reported preferential substrate for Cas13 trans-cleavage activity. ^[20,61]^

**Table S3. Sequences used in MARPLE version detecting Anti-DIG Antibdoy**

| **Name** | **Sequence (5’- 3’)** | Figure |
| --- | --- | --- |
| DCD1_th2 | *DIG- TTCTCCA****AG****TCAACATCAGTCTGATAAGCTA* | 2B |
| DCD2_th2 | *DIG- TTCTCCAATCAGACTGATGTTGA****CT*** | 2B |
| DCD1_th3 | *DIG-TTCTCCA****CAG****TCAACATCAGTCTGATAAGCTA* | 2-4, Supplementary Figures S2-S8 |
| DCD2_th3 | *DIG-TTCTCCAATCAGACTGATGTTGA****CTG*** | 2-4, Supplementary Figures S2-S8 |

Here are listed DNA sequences used in anti-DIG antibody detection system. DIG-conjugated DNA sequences (DCD1 and 2) present toehold portion (th) of increase length in **bold**.

**Table S4. Sequences used in MARPLE version detecting Anti-CH Antibody**

| **Name** | **Sequence (5’- 3’)** | Figure |
| --- | --- | --- |
| CDC1_th3 | *CH- TTCTCCA****CAG****TCAACATCAGTCTGATAAGCTA* | Supplementary Figure S9 |
| CDC2_th3 | *CH- TTCTCCAATCAGACTGATGTTGA****CTG*** | Supplementary Figure S9 |

DNA sequences used in anti-CH antibody detection system. CH-conjugated DNA sequences (CDC1 and 2) present toehold portion (th) in **bold**.

**Table S5. Sequences used in MARPLE version detecting Trastuzumab**

| **Name** | **Sequence (5’- 3’)** | **Figure** |
| --- | --- | --- |
| Peptide-PNA Antigen Trastuzumab | *TCTTATTTTGCGGTGAC–N-term–QLG PYE LWE LSH–C-term* | 5 A-E, Supplementary Figure S10 |
| TD1_th3 | *GTCACCGCAAAATAAGATTCTCCA****CAG****TCAACATCAGTCTGATAAGCTA* | 5 A-E, Supplementary Figure S10 |
| TD2_th3 | *GTCACCGCAAAATAAGATTCTCCAATCAGACTGATGTTGA****CTG*** | 5 A-E, Supplementary Figure S10 |

DNA sequences used in Trastuzumab detection system. Trastuzumab DNA sequences (TD1 and TD2) present the toehold portion (th) in bold.

**Table S6. Sequences used in MARPLE version detecting anti-HA**

| **Name** | **Sequence (5’- 3’)** | **Figure** |
| --- | --- | --- |
| Peptide-PNA Antigen anti-HA | *N-term-YPYDVPDYA-C-term-CAGTGGCGT* | 5 A, 5F-I, Supplementary Figure S13 |
| HAD1_th3 | *ACGCCACTGTTCTCCA****CAG****TCAACATCAGTCTGATAAGCTA* | 5 A, 5F-I, Supplementary Figure S13 |
| HAD2_th3 | *ACGCCACTGTTCTCCAATCAGACTGATGTTGA****CTG*** | 5 A, 5F-I, Supplementary Figure S13 |

In the above sequences, the DNA sequences used in anti-HA antibody detection system. Anti-HA DNA sequences (HAD1 and HAD2) present the toehold portion (th) in **bold**.

**Table S7. Sequences used in MARPLE version detecting anti-MUC1**

| **Name** | **Sequence (5’- 3’)** | **Figure** |
| --- | --- | --- |
| MDC_s1 | DBCO-TTTTTTTTTTTTCTCCA**CAG**TCAACATCAGTCTGATAAGCT | 6, Supplementary Figure S16 |
| MDC_s2 | DBCO-TTTTTTTTTTTTCTCCAATCAGACTGATGTTGA**CTG** | 6, Supplementary Figure S16 |

In the above sequences, the **bold** bases indicate the toehold.

**Table S8. Sequences used in ELISA experiments**

| **Name** | **Sequence (5’- 3’)** | **Figure** |
| --- | --- | --- |
| HA Peptide | N-term MEYPYDVPDYAAEYPYDVPDYAAEYPYDVPDYAAKLE-C-term | S14 |
| PNA-MUC1 Peptide | ACT AAA TTC GAC TAT CC–3’ N term - APDTRPAPGSTAPPA -C term | S17 |

**Supplementary Figures**


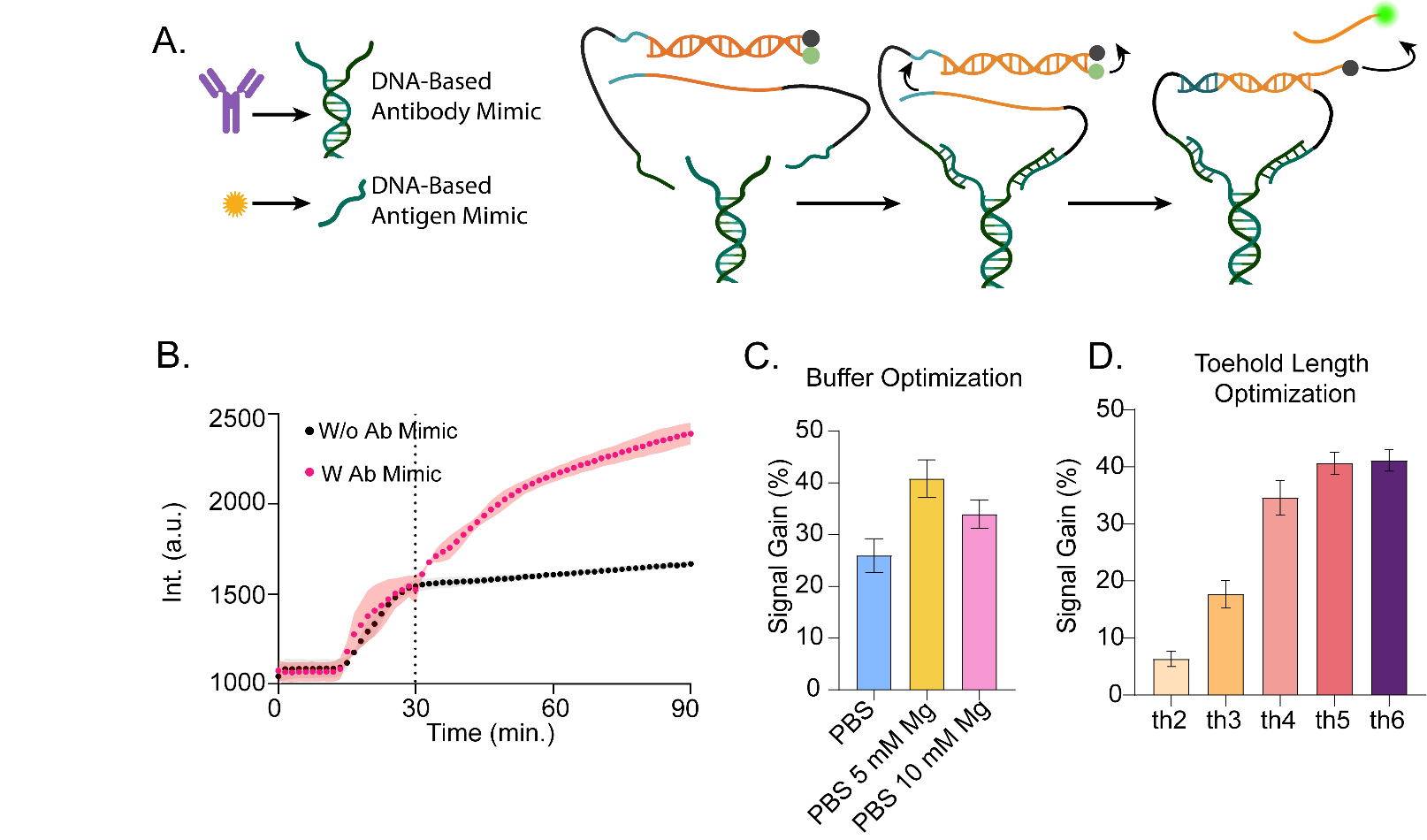
**Figure S1**. A) Schematic of the DNA-based model system used to evaluate the proximity-triggered toehold exchange reaction. B) Fluorescence kinetics of the toehold-exchange reaction triggered by an antibody mimic (AB-mimic), which was added at minute 30.). C) Optimization of Mg2+ concentration in the reaction buffer to maximize strand exchange efficiency. D Evaluation of toehold length on exchange reaction efficiency. Error bars and shaded regions represent standard deviation across three independent replicates (n=3).


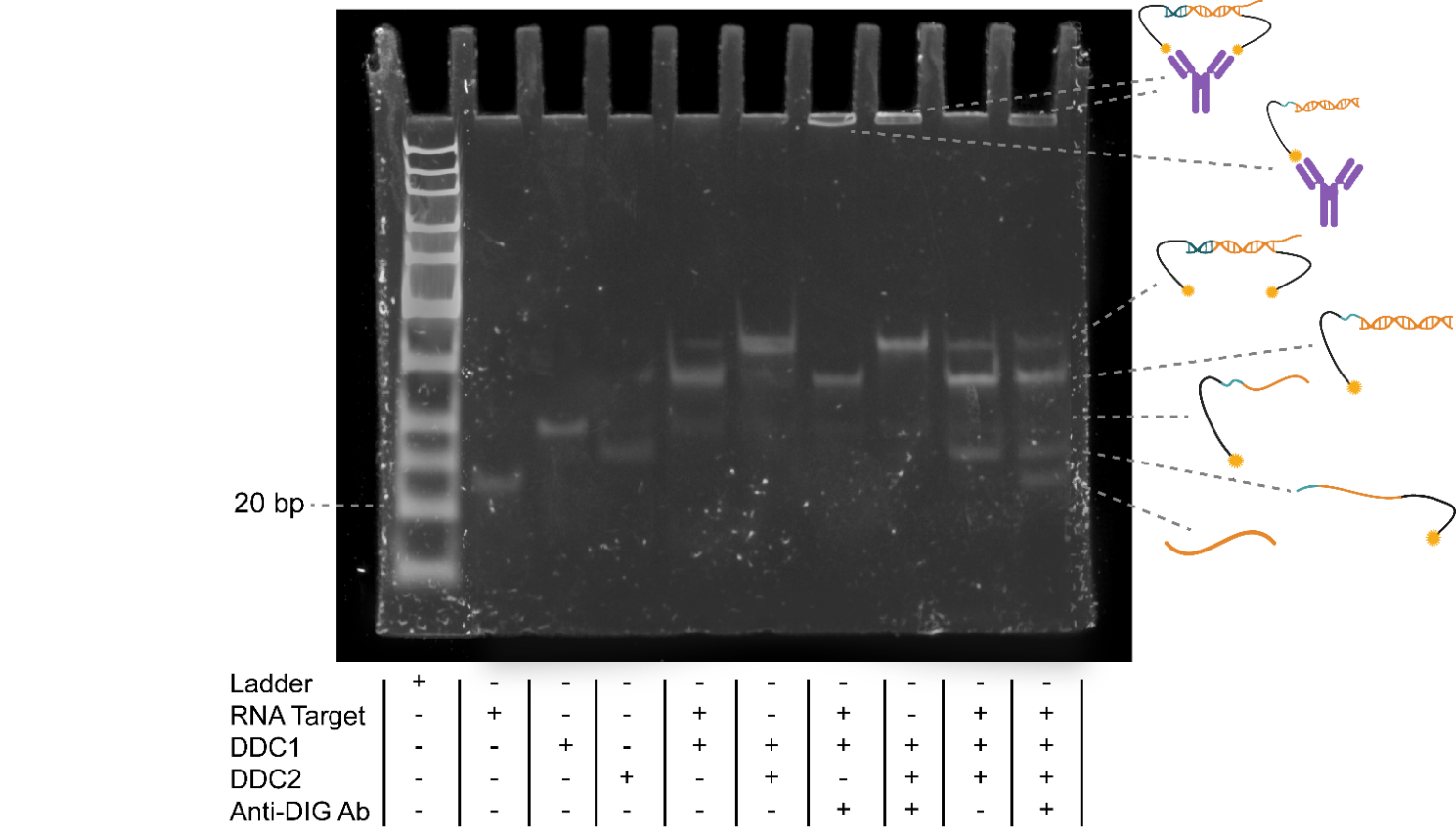


**Figure S2.** Native 10% acrylamide gel showing anti-DIG-triggered toehold-exchange reaction. Lane 1: DNA ladder; Lane 2: RNA Target (22 bp); Lane 3: DDC1; Lane 4: DDC2; Lane 5: ADC1-RNA target heteroduplex; Lane 6: ADC1-ADC2 homoduplex; Lane 7: DDC1-RNA Target heteroduplex in the presence of anti-DIG antibody; Lane 8: DDC1-DDC homoduplex in the presence of anti-DIG antibody; Lane 9: ADC1-RNA Target heteroduplex in the presence of ADC2 (i.e. the blank sample in the absence of antibody); Lane 10: ADC1-RNA Target heteroduplex in the presence of ADC2 and anti-DIG antibody.


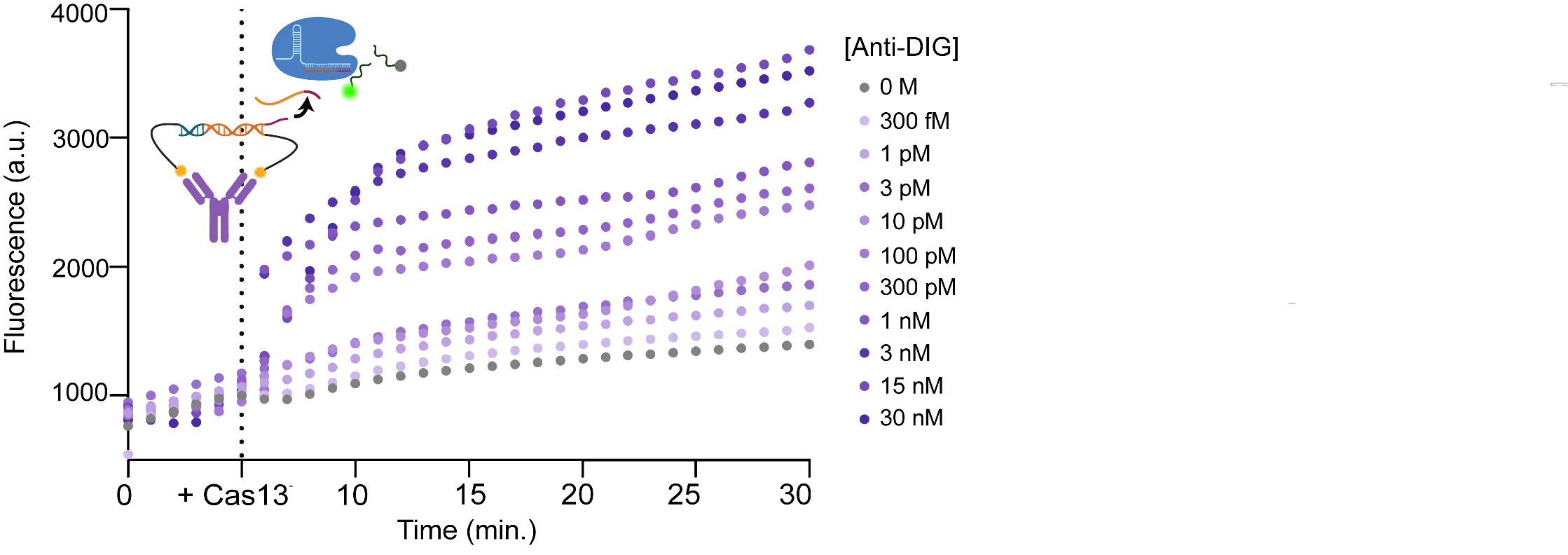
 **Figure S3.** Fluorescence kinetics measurements for the anti-DIG MARPLE platform in buffer at varying concentrations of target antibody. The experiments were conducted in PBS supplemented with MgCl_2_ 5mM, with fixed concentrations of nucleic acid components (5 nM), Cas13 (30 nM), and RNA hairpin reporter (100 nM) at 25° C. Error bars represent standard deviation across three independent replicates (n=3).

**Figure S4.** A) Stability of RNA hairpin reporters in 10%,20%, 50% and 75% human serum in PBS supplemented with 5mM MgCl_2_. The RNA hairpin reporter (100 nM) was incubated in the different serum concentrations, and reporter integrity was monitored over 30 minutes via fluorescence kinetic measurements. B) Stability of ADC1-Target heteroduplex in 10%,20%, 50% and 75% human serum in PBS supplemented with 5mM MgCl_2_. The heteroduplex was pre-formed in PBS supplemented with 5mM MgCl_2_ with annealing reaction. A total of 30 nM heteroduplex was incubated in the different serum concentrations, and heteroduplex integrity was monitored over 30 minutes via fluorescence kinetic measurements. C) Fluorescence kinetics measurements in 10% serum diluted in PBS supplemented with 5mM MgCl_2_ for the anti-DIG MARPLE platform in buffer at varying concentrations of target antibody. D) Optimization of Mg²⁺ concentration for Cas13 trans-cleavage activity in serum. Cas13 (30 nM) activated by RNA target (10 nM) was incubated in a reaction mixture containing 10% human serum supplemented with varying concentrations of Mg²⁺, and trans-cleavage activity was monitored over 25 minutes. Endpoint fluorescence intensities were used to calculate signal gain %. Error bars represent standard deviation from three independent replicates.


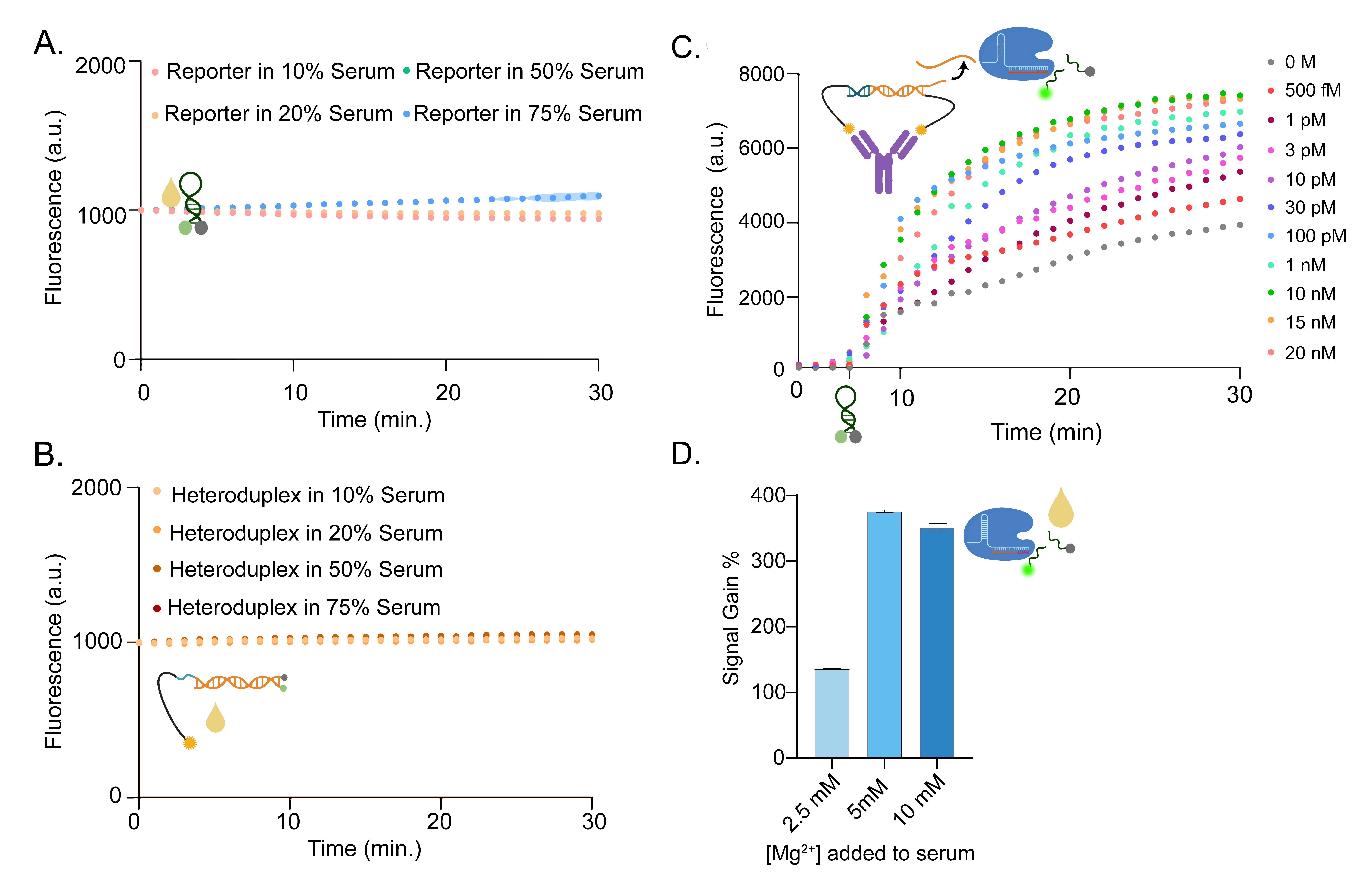


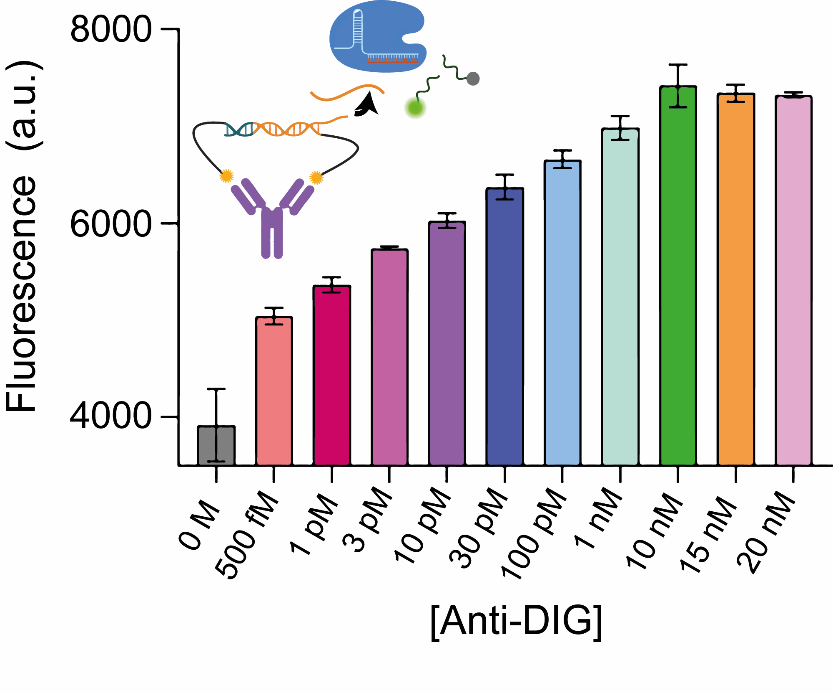
F**igure S5.** Fluorescence intensities for MARPLE as a function of increasing anti-DIG concentration. The experiments were conducted in 10% serum in PBS supplemented with MgCl_2_ at a final concentration of 5mM, with fixed concentrations of nucleic acid components (5 nM), Cas13 (30 nM), and RNA hairpin reporter (100 nM) at 25° C. Error bars represent standard deviation across three independent replicates (n=3).

**Figure S6.** Off-target assessment of MARPLE. The figure reports signal gains from MARPLE-based detection of 100 pM anti-Dig antibody compared with analogous systems but containing random RNA sequences in the place of the correct RNA target. All experiments were conducted using 5 nM of nucleic acid components, 30 nM Cas13, and 100 nM RNA hairpin reporter at 25°C in 10% serum diluted in PBS supplemented with 5mM MgCl_2_. Signal gain (%) was calculated relative to background fluorescence. Error bars represent standard deviation across three independent replicates (n=3).


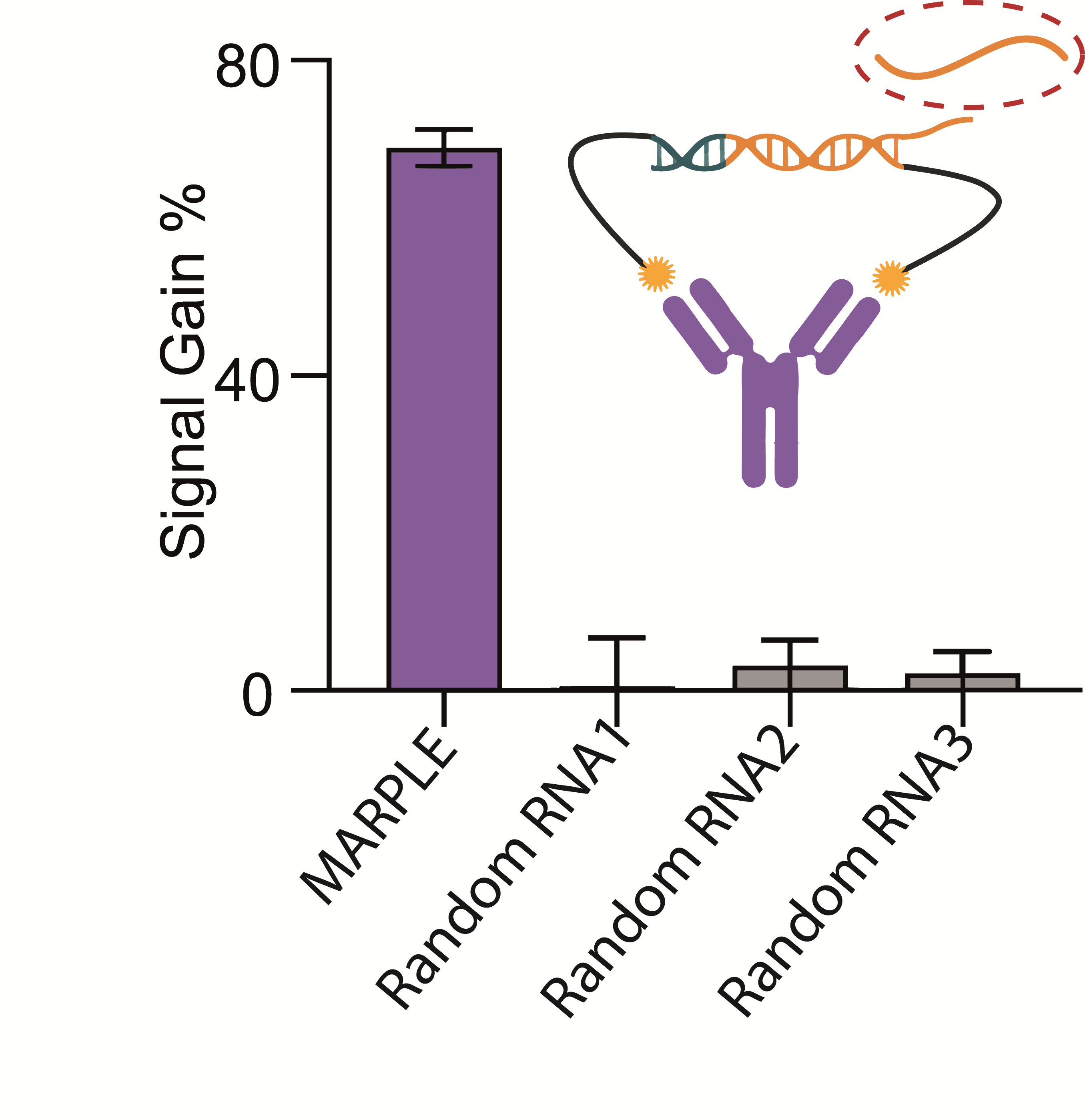


**
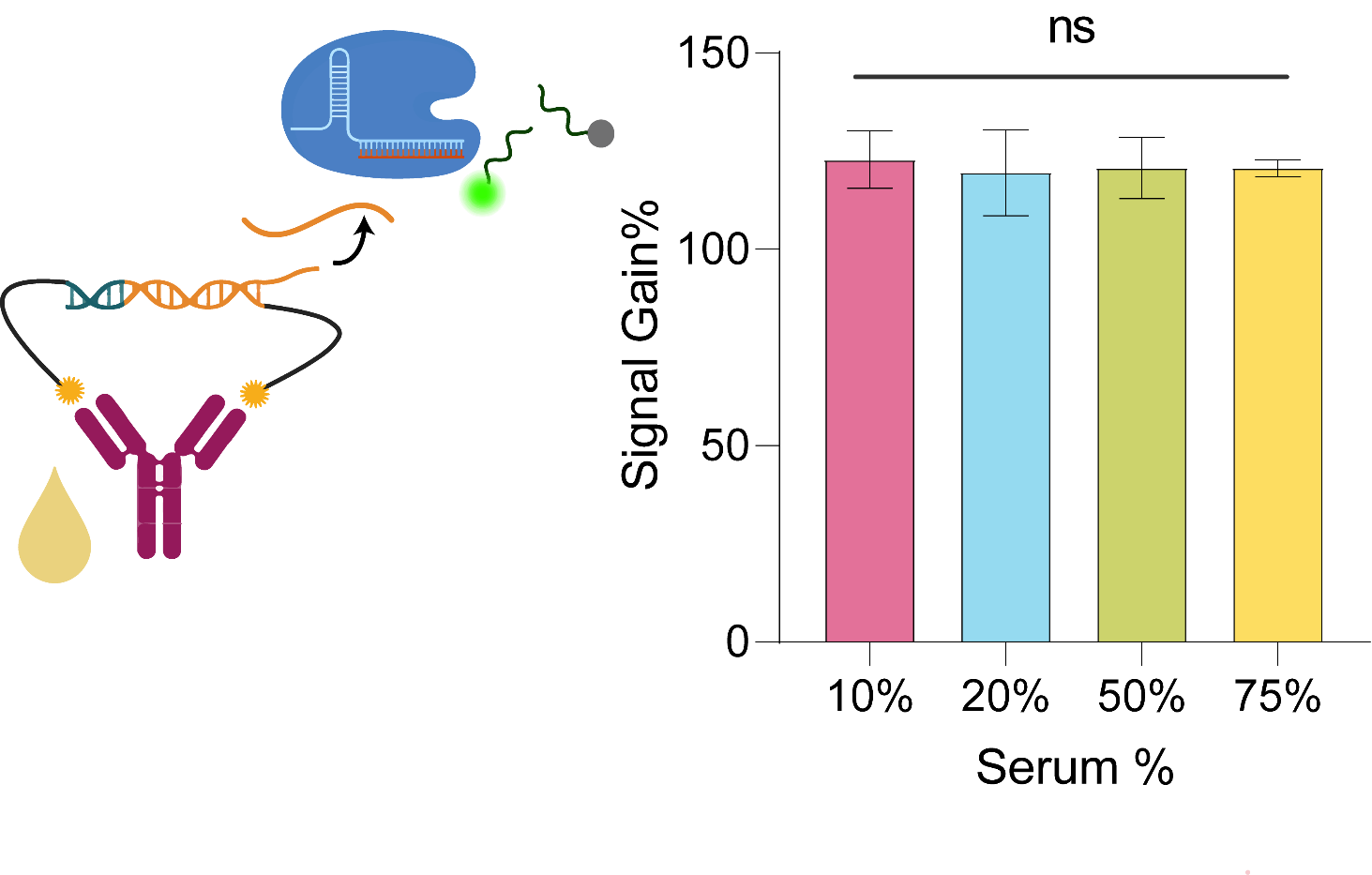
**

**Figure S7**. Performance of MARPLE across varying serum concentrations for 10 nM Anti-DIG antibody. Human serum was diluted in PBS, with a final concentration of 5mM MgCl_2_. Signal Gain (%) was calculated relative to background signal from the same serum conditions without antibody. No significant difference was observed across serum concentrations (One-Way ANOVA, P = 0.9915). Error bars represent standard deviation from three independent replicates (n=3), with relative standard deviation (RSD) of 10% at 10% serum, 16% at 20% serum, 11% at 50% serum and 3% at 75% serum.


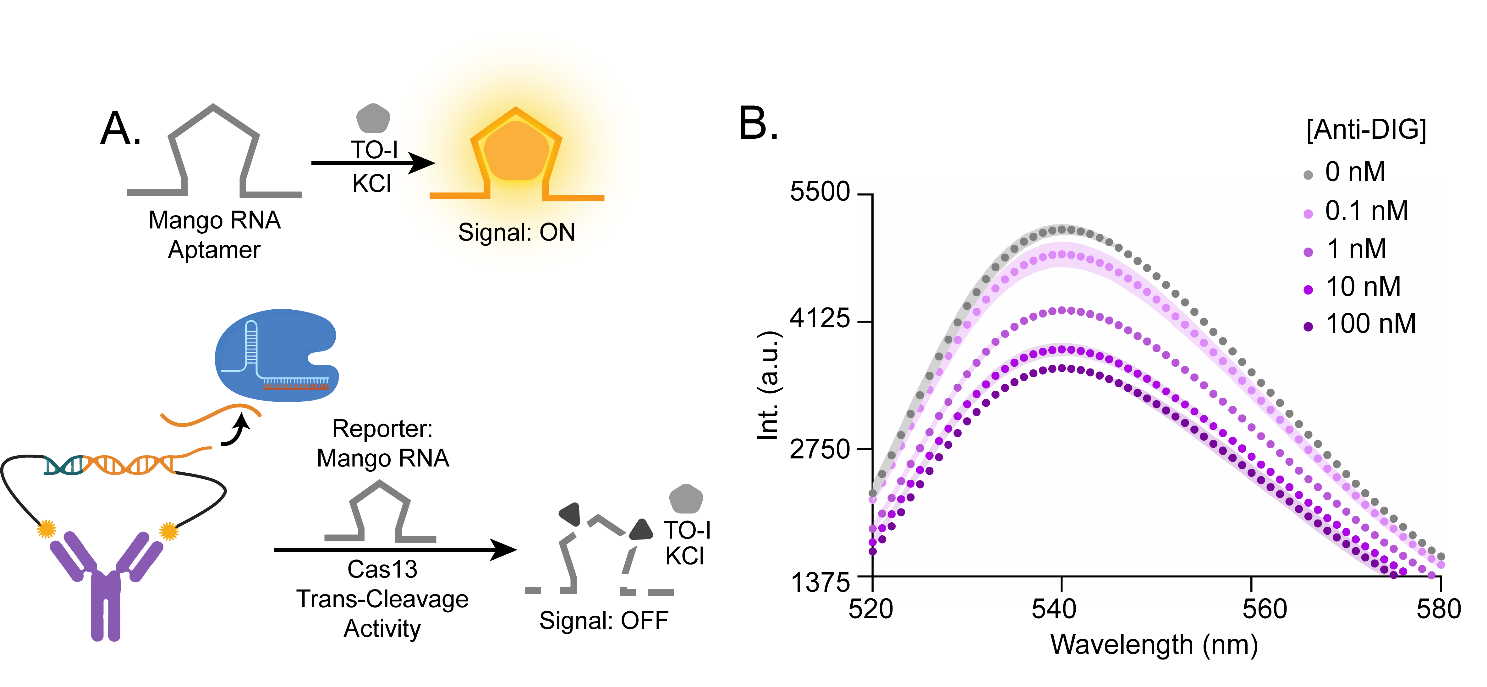
**Figure S8.** A) Schematic overview of the MARPLE assay using Mango RNA as the reporter. Upon RNA target release and Cas13 activation, collateral cleavage of Mango RNA prevents fluorophore binding, generating a signal-off response. B) Fluorescence response curves obtained using varying concentrations of anti-DIG antibody buffer. Samples contained 5 nM nucleic acid components (DDC1–RNA target heteroduplex and DDC2), incubated with different concentrations of anti-DIG antibody for 15 minutes. Cas13 (30 nM) and Mango RNA (100 nM) were then added, and fluorescence spectra were recorded after 25 minutes. Shaded regions represent standard deviation across three independent replicates (n=3).


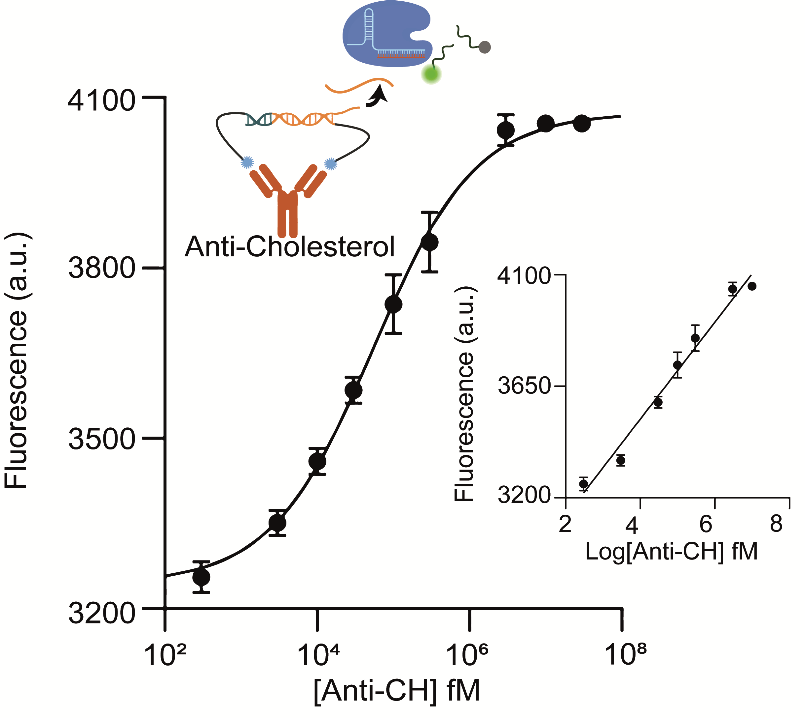

**Figure S9**: Dose-response curve showing increase in fluorescence as a function of anti-CH antibody concentration. Inset: calibration curve obtained by linear fitting of endpoint fluorescence intensity values in the 300 fM –3 nM anti-CH range (LOD=212 fM). The curve is described by the following equation: Y = 195.7*X + 2735, R^2^ = 0.971. The experiments were conducted in buffer with fixed concentrations of nucleic acid components (5 nM), Cas13 (30 nM), and RNA hairpin reporter (100 nM) at 25° C. Error bars represent standard deviation across three independent replicates (n=3).


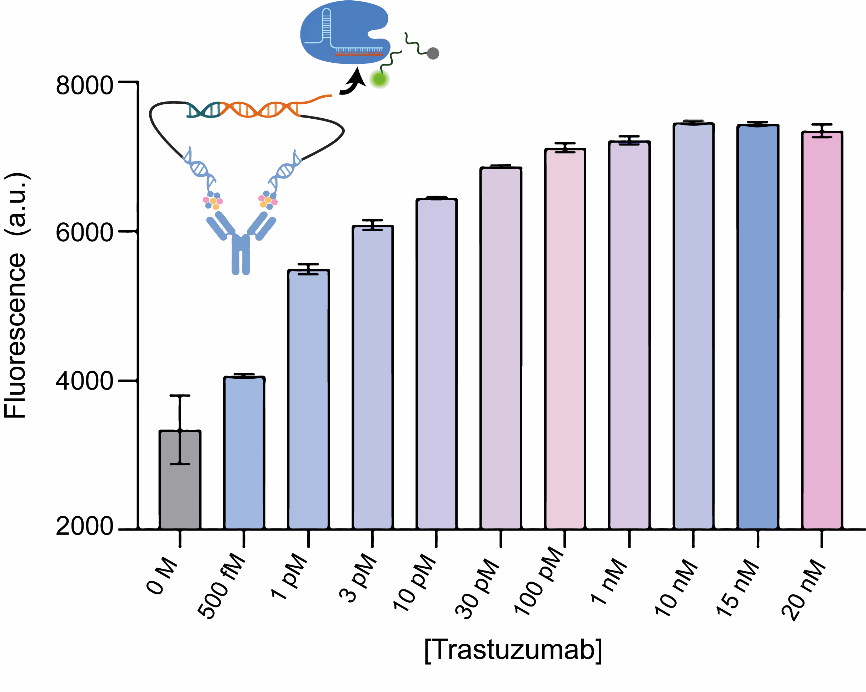
**Figure S10.** Fluorescence intensities for MARPLE as a function of increasing Trastuzumab concentration in 10% serum. The experiments were conducted in 10% serum in PBS supplemented with MgCl_2_ at a final concentration of 5mM, with fixed concentrations of nucleic acid components (5 nM), Cas13 (30 nM), and RNA hairpin reporter (100 nM) at 25° C. Error bars represent standard deviation across three independent replicates (n=3).

**Figure S11**. A) Schematic representation of the sandwich ELISA for Trastuzumab detection. B) Binding curve for Trastuzumab quantification, obtained by fitting absorbance values at 450 nm (UAbs₄₅₀nm) as a function of Trastuzumab concentration. The curve demonstrates a typical sigmoidal dose–response, described by a Langmuir-type equation: y= 0.068 + [([Trastuzumab] * (0.305 - 0.053))/ ([Trastuzumab] + 1.595)]. C) Recovery % of Trastuzumab from spiked samples at different concentrations (5, 15, and 50 nM). The error bars represent standard deviation across three independent replicates (n=3).


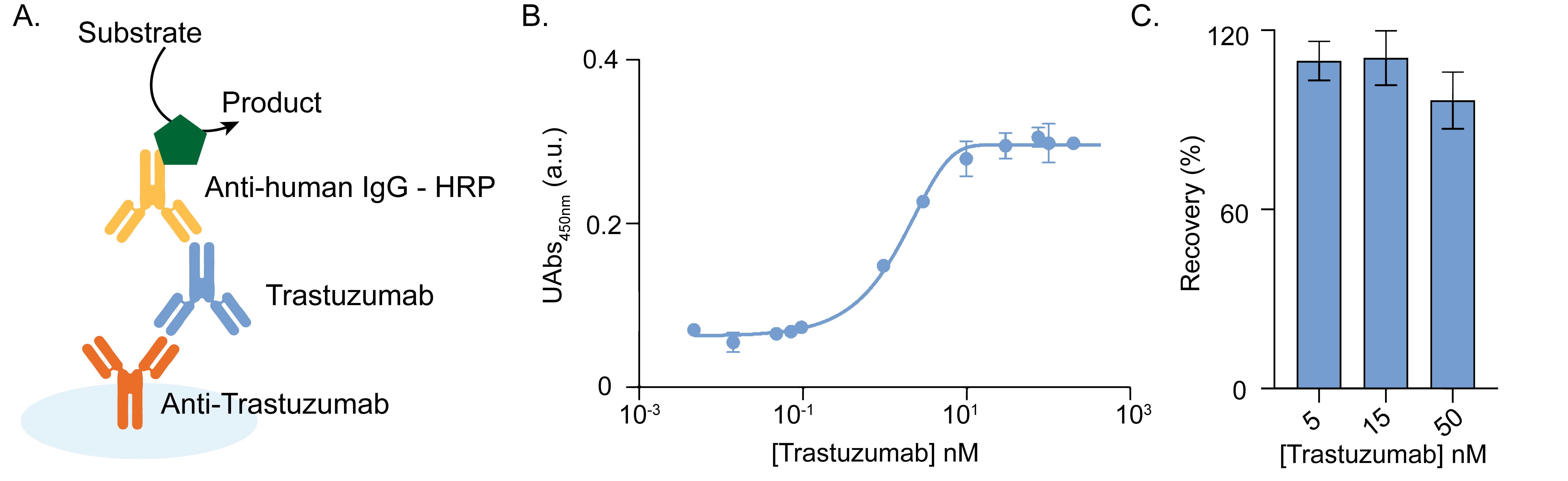


**
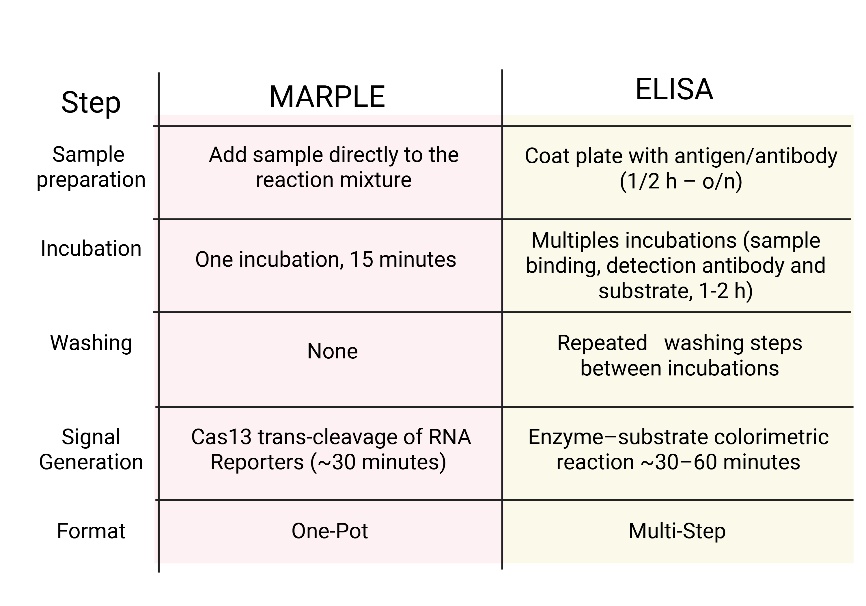
Figure S12.** Side-by-side comparison of MARPLE and conventional ELISA workflows.

F**igure S13.** Fluorescence intensities for MARPLE as a function of increasing anti-HA concentration in 10% serum. The experiments were conducted in 10% serum in PBS supplemented with MgCl_2_ at a final concentration of 5mM, with fixed concentrations of nucleic acid components (5 nM), Cas13 (30 nM), and RNA hairpin reporter (100 nM) at 25° C.
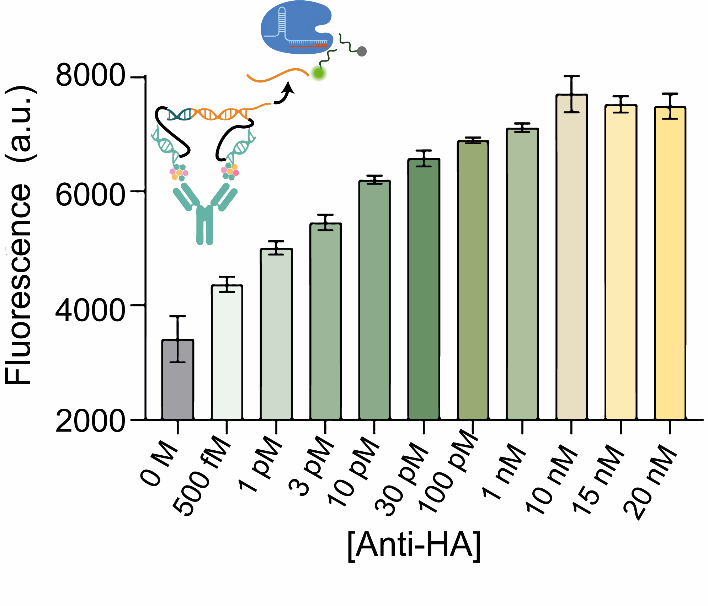
 Error bars represent standard deviation across three independent replicates (n=3).

**Figure S14**. A) Schematic representation of the sandwich ELISA for anti-HA detection. B) Binding curve for anti-HA quantification, obtained by fitting absorbance values at 450 nm (UAbs₄₅₀nm) as a function of anti-HA concentration. The curve demonstrates a typical sigmoidal dose–response, described by a Langmuir-type equation: y= -0.026 +[([anti-HA] * (2.179 - (-0.026)))/ ([anti-HA] + 1.161)]]. C) Recovery % of anti-HA from spiked samples at different concentrations (0.3, 1, and 3 nM). The error bars represent standard deviation across three independent replicates (n=3).


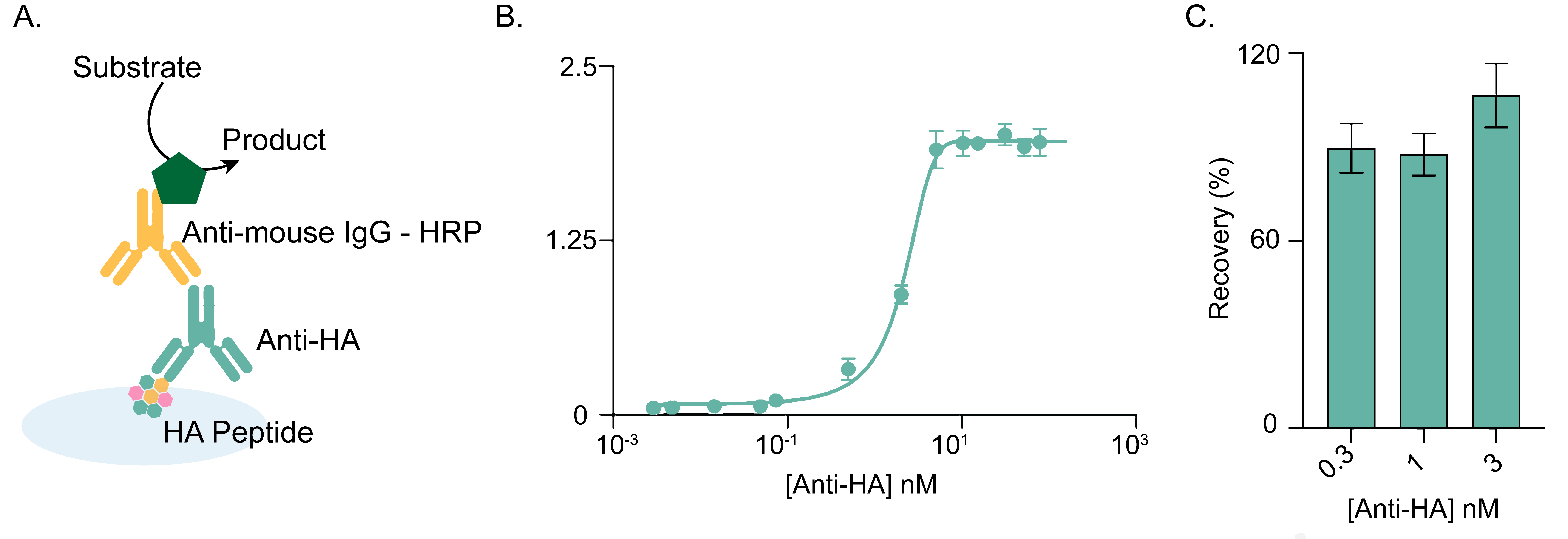


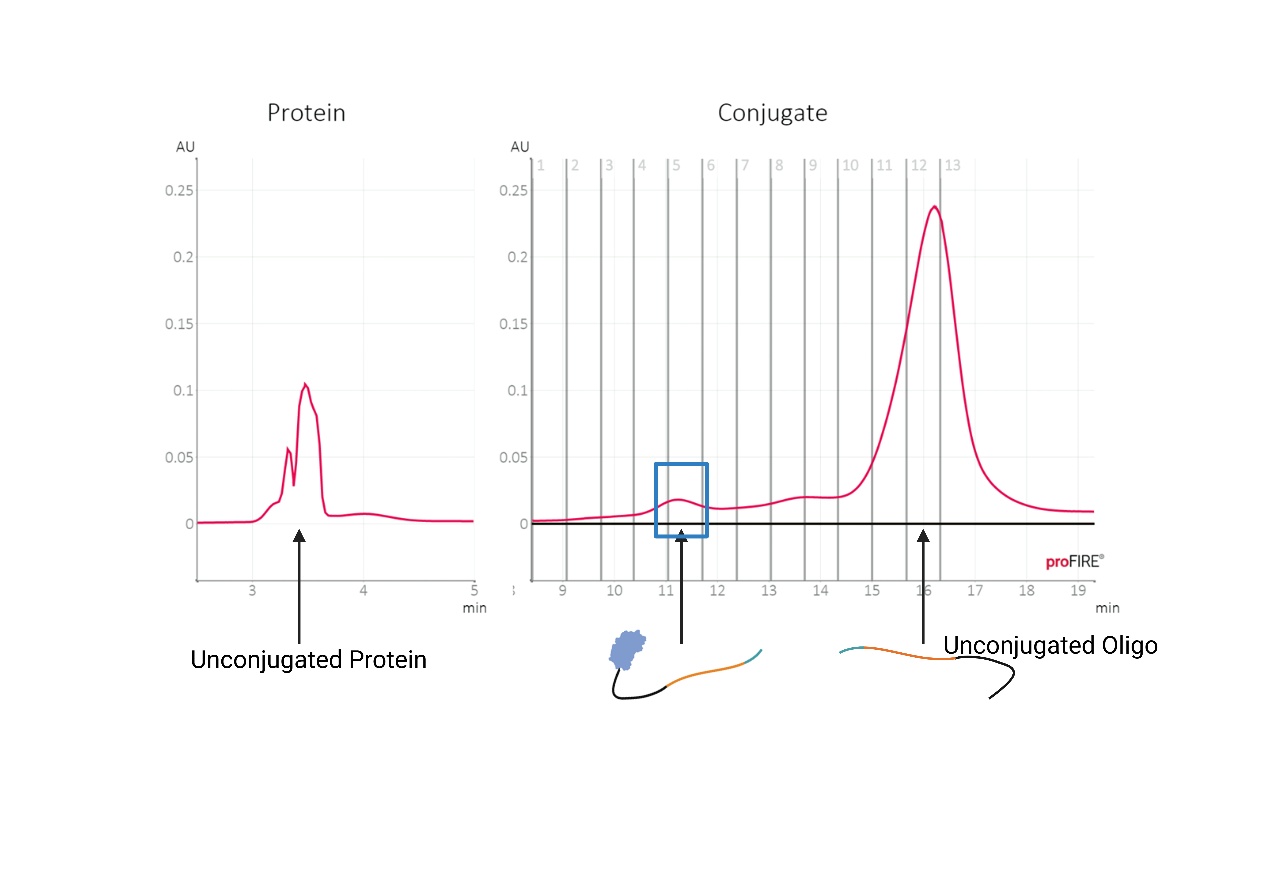

**Figure S15**. proFIRE® output purification chromatograms of ADC1-MUC1 conjugate. The chromatogram shows a first peak corresponding to unconjugated MUC1, a second peak for the conjugation product, and a final peak for unreacted oligonucleotide.

F**igure S16.** Fluorescence intensities for MARPLE as a function of increasing anti-MUC1 concentration in 10% serum. The experiments were conducted in 10% serum in PBS supplemented with MgCl_2_ at a final concentration of 5mM, with fixed concentrations of nucleic acid components (5 nM), Cas13 (30 nM), and RNA hairpin reporter (100 nM) at 25° C.
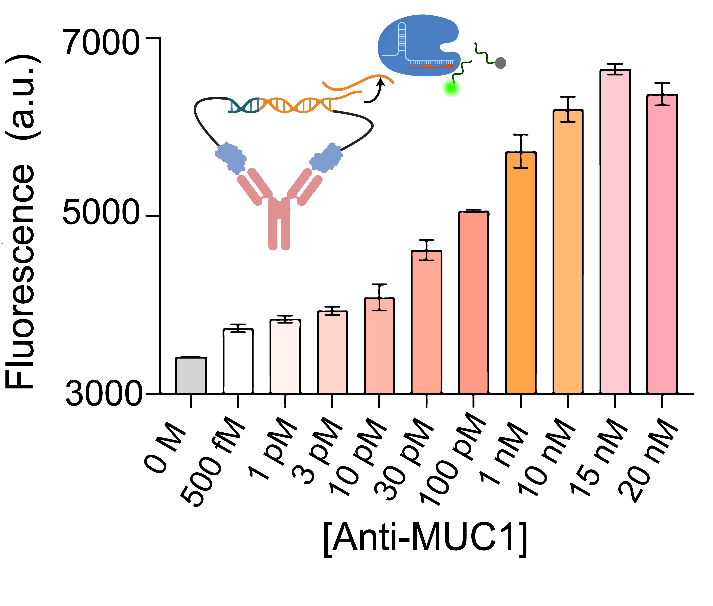
 Error bars represent standard deviation across three independent replicates (n=3).

**Figure S17**. A) Schematic representation of the sandwich ELISA for anti-MUC1 detection. B) Binding curve for anti-MUC1 quantification, obtained by fitting absorbance values at 450 nm (UAbs₄₅₀nm) as a function of anti-HA concentration. The curve demonstrates a typical sigmoidal dose–response, described by described by a standard Langmuir equation: y= 0.048 + [([anti-MUC1] * (0.233 - 0.048))/ ([anti-MUC1] + 18.30)]. C) Recovery % of anti-MUC1 form spiked samples at different concentrations (5, 15, and 50 nM). The error bars represent standard deviation across three independent replicates (n=3).


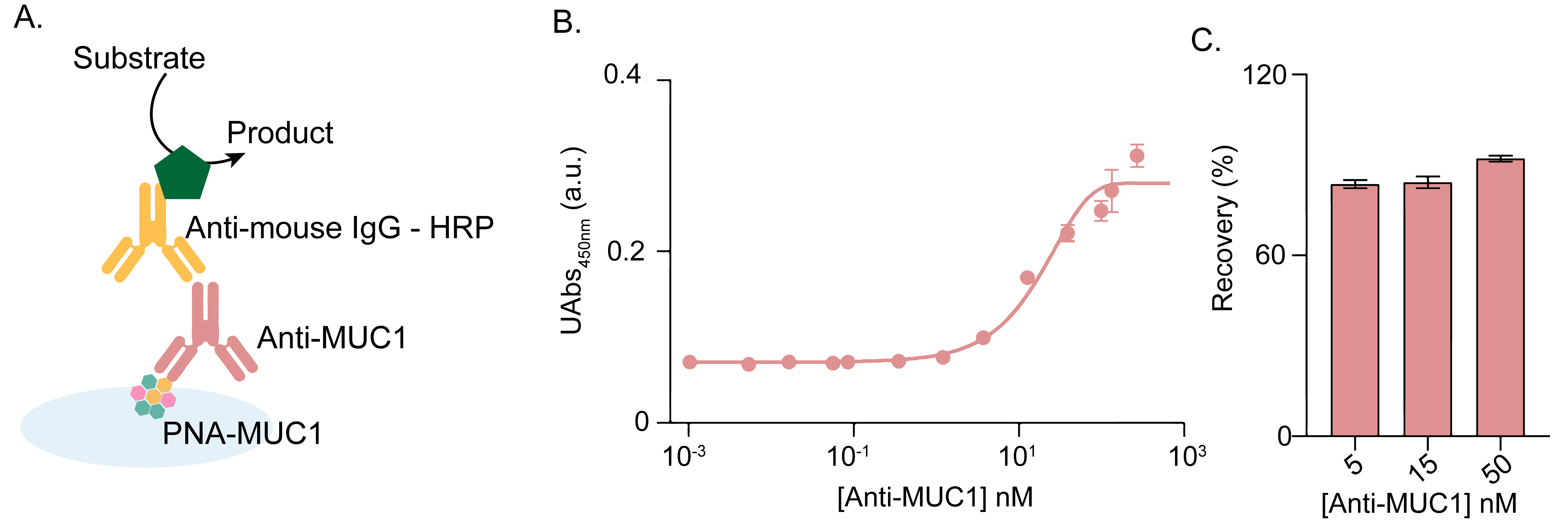


**Supplementary References**

[59] N. Zadeh, C. D. Steenberg, J. S. Bois, B. R. Wolfe, M. B. Pierce, A. R. Khan, R. M. Dirks, N. A. Pierce, J. Comput. Chem. 2011, 32, 170-173.

[60] R. Owczarzy, A. V. Tataurov, Y. Wu, J. A. Manthey, K. A. McQuisten, H. G. Almabrazi, K. F. Pedersen, Y. Lin, J. Garretson, N. O. McEntaggart, C. A. Sailor, R. B. Dawson, A. S. Peek, Nucleic Acids Res. 2008, 36, W163-W169.

[61] J. S. Gootenberg, O. O. Abudayyeh, M. J. Kellner, J. Joung, J. J. Collins, F. Zhang, Science. 2018, 360(6387), 439-444.

[62] T. Scientific. General Sandwich ELISA Protocol. https://www.thermofisher.com/it/en/home/references/protocols/cell-and-tissue-analysis/elisa-protocol/general-elisa-protocol.html (Accessed 10.07.25)
